# Bifunctional effects of O-methylated flavones from *Scutellaria baicalensis* Georgi on melanocytes: Inhibition of melanin production and intracellular melanosome transport

**DOI:** 10.1371/journal.pone.0171513

**Published:** 2017-02-09

**Authors:** Michiko Kudo, Kumiko Kobayashi-Nakamura, Kentaro Tsuji-Naito

**Affiliations:** Fundamental Research Laboratory, Fundamental Research Department, DHC Corporation, Chiba, Japan; University of Alabama at Birmingham, UNITED STATES

## Abstract

The growing interest in skin lightening has recently renewed attention on the esthetic applications of Chinese herbal medicine. Although *Scutellaria baicalensis* Georgi is used for antipyretic and antiinflammatory purposes, its whitening effect remains unclear. This study reports three major findings: (1) *S*. *baicalensis* has a potent inhibitory effect on melanogenesis; (2) wogonin and its glycoside are the active components of *S*. *baicalensis*; and (3) *O*-methylated flavones from *S*. *baicalensis*, such as wogonin, inhibit intracellular melanosome transport. Using a melanin quantification assay, we showed that *S*. *baicalensis* potently inhibits melanogenesis in B16F10 cells. Componential analyses revealed that the main components of *S*. *baicalensis* are baicalin, wogonoside, baicalein, wogonin, and oroxylin A. Among these five flavones, wogonin and wogonoside consistently inhibited melanogenesis in both B16F10 melanoma cells and primary melanocytes. Wogonin exhibited the strongest inhibition of melanin production and markedly lightened the color of skin equivalents. We identified microphthalmia-associated transcription factor and tyrosinase-related proteins as potential targets of wogonin- and wogonoside-induced melanogenesis suppression. In culture, we found that the melanosomes in wogonin-treated B16F10 cells were localized to the perinuclear region. Immunoblotting analyses revealed that wogonin significantly reduced in melanophilin protein, which is required for actin-based melanosome transport. Other actin-based melanosome transport-related molecules, i.e., Rab27A and myosin Va, were not affected by wogonin. Cotreatment with MG132 blocked the wogonin-induced decrease in melanophilin, suggesting that wogonin promotes the proteolytic degradation of melanophilin via the calpain/proteasomal pathway. We determined that the structural specificities of the mono-*O*-methyl group in the flavone A-ring and the aglycone form were responsible for reducing melanosome transport. Furthermore, wogonin and two wogonin analogs, mono-*O*-methyl flavones, strongly suppressed melanosome transport. Our findings suggest the applicability of *S*. *baicalensis* in the esthetic field. Thus, we propose a novel pharmacologic approach for the treatment of hyperpigmentation.

## Introduction

Melanocytes in the epidermis are responsible for producing melanin, a black pigment that protects against ultraviolet radiation [1]. Melanocytes synthesize numerous secretory melanosomes containing proteins and melanin, which are then transferred to neighboring keratinocytes [2]. Prior to release from melanocytes, melanosomes move from the perinuclear region to the cell periphery through melanization by enzymes required for melanin synthesis, including tyrosinase (TYR), tyrosinase-related protein 1 (TYRP1), and dopachrome tautomerase (DCT) [3]. These melanogenetic enzymes are transcriptionally regulated by microphthalmia-associated transcription factor (MITF), which is a master transcription factor of melanocyte development, differentiation, and survival. The intercellular transport of melanosomes requires several steps, including bidirectional long-range transport on microtubules to the apical surface, transfer to actin filaments, irreversible short-range transport by actin dynamics, and binding to the cytomembrane [4].

Several independent researchers recently reported that Rab27A, synaptotagmin-like protein (SLP) 2A/synaptotagmin 2, melanophilin (MLPH)/SLP homolog lacking C2 domains-A, and myosin Va are involved in the regulation of melanosome transport [4, 5]. Rab27A and myosin Va interact via MLPH to form a tripartite complex, which serves as a regulator of actin-based transport. Rab27A links SLP2A with phosphatidylserine, thereby docking melanosomes with the apical plasma membrane. This finding suggests that SLP2A is a regulator of melanosome exocytosis. Mutations in genes encoding melanosome transport-related molecules, such as myosin Va, Rab27A, and MLPH, cause a human pigmentary disease known as Griscelli syndrome, which is associated with diluted skin and hair color [6–8]; therefore, these molecules play an important role in modulating skin color variations.

*Scutellaria baicalensis* Georgi is a perennial herb belonging to the Lamiaceae family. Its root has been used as a traditional Chinese medicine to alleviate inflammation, allergy, and fever as well as a treatment for several cancers [9, 10]. Various bioactive components, such as flavonoids, have been identified in *S*. *baicalensis* root parts. Baicalin, baicalein, wogonin, and wogonoside are the main components of the root, and all these compounds have significant pharmacological effects [11]. In recent years, the whitening effect of dried *S*. *baicalensis* root has attracted significant attention from the Asian cosmetic field; however, a comprehensive study on the whitening effect of *S*. *baicalensis*, including its mechanism of action, has not been conducted. Therefore, we studied the functional link between the whitening effect of *S*. *baicalensis* and the antimelanogenic effects of its major flavonoids. Our results identified wogonin and its glycoside as the most potent regulatory constituents of *S*. *baicalensis*. In addition, we showed the bifunctionality of wogonin, including the downregulation of both melanin production and intracellular melanosome transport. In this study, we provide plausible evidence that both the aglycone form and the *O*-methyl group on the flavone A-ring likely contribute to the inhibition of intracellular melanosome transport. Thus, our study provides a better pharmacologic understanding of flavones as depigmenting agents.

## Materials and methods

### Materials

Baicalin, baicalein, and wogonin were purchased from Wako Pure Chemical (Osaka, Japan). Wogonoside and oroxylin A were purchased from Faces Biochemical (Daejeon, Korea). Norwogonin was purchased from Extrasynthese (Lyon, France). H-89 and anti-glyceraldehyde-3-phosphate dehydrogenase (GAPDH, CB1001) antibodies were obtained from Calbiochem (La Jolla, CA, USA). 7-*O*-Methyl baicalein was purchased from Indofine (Hillsborough, NJ, USA), and rapamycin was purchased from LKT Laboratories (St. Paul, MN, USA). Anti-MLPH (ab2716) and anti-Rab27A (ab55667) antibodies were purchased from Abcam (Cambridge, MA, USA), and anti-TYR (M-19, sc7834), anti-TYRP1 (G-17, sc10443), anti-DCT (D-18, sc10451), and horseradish peroxidase (HRP)-conjugated anti-goat antibodies were obtained from Santa Cruz Biotechnology (Santa Cruz, CA, USA). HRP-conjugated anti-mouse antibodies were obtained from GE Healthcare (Chicago, IL, USA). Anti-myosin Va antibody (#3402) was purchased from Cell Signaling Technology (Danvers, MA, USA). Anti-MITF (MS-771-P0), Alexa Fluor 594 phalloidin, and Alexa Fluor 488-conjugated anti-goat antibodies were obtained from Thermo Fisher Scientific (Waltham, MA, USA). MG132 and leupeptin were purchased from Sigma-Aldrich (St. Louis, MO, USA). DAPI was purchased from Dojindo Laboratories (Kumamoto, Japan), and linoleic acid (LA) was purchased from Tokyo Chemical Industry (Tokyo, Japan).

### Preparation of *S*. *baicalensis* root extract

Dried chips of *S*. *baicalensis* root were obtained from a commercial supplier. Chips were ground in a mortar to a coarse powder. To prepare a crude extract from *S*. *baicalensis* root, the powder (0.51 g) was added to 10 ml of 70% ethanol and left to stand at room temperature for 24 h after sonication. The supernatant was fractionated using a Pasteur pipette and then filtered through a membrane filter (0.45 μm). The resultant solution was concentrated by rotary evaporation. After subsequent standing in a desiccator for 4 days, the crude extract (27.4 mg) was obtained. Using the same procedure, the organic solvents of n-hexane, ethylacetate, methanol (MeOH), and water resulted in 83.2, 109.2, 177.6, and 84.4 mg of the crude extract from the ratio of powder/solvent (20.3 g/100 ml, 10.1 g/50 ml, 1.0 g/5 ml, and 1.0 g/30 ml), respectively. For extraction with water, the filtrate was freeze-dried for 6 days. For the fractionated samples, the crude MeOH extract (2.3 g) was prepared from 10 g of dried powder in 150 ml of MeOH. Next, 1.0 g of the crude MeOH extract was passed through a Sep-Pak C18 cartridge (Waters, Milford, MA, USA) and eluted stepwise with a series of MeOH solutions, including water, 25%, 50%, 75%, and 100% MeOH. After solid-phase extraction, each fraction was freeze-dried after vaporization. We obtained 889.7, 40.7, 111.5, 46.1, 28.0 mg of fractional residue from water, 25%, 50%, 75%, and 100% MeOH, respectively. The test samples of each extraction were reconstituted with distilled water, MeOH, or dimethyl sulfoxide to generate the crude extract and fractionated solutions. Each extraction solution was aliquoted and stored at –20°C until further use. Once dissolved for testing, each extraction solution was stored at 4°C and used within one week.

### Cell cultures

The mouse melanoma cell line B16F10 was provided by the Cell Resource Center for Biomedical Research, Tohoku University (Miyagi, Japan). B16F10 cells were maintained as monolayer cultures in Dulbecco’s modified Eagle medium (DMEM) supplemented with 10% fetal bovine serum (FBS) at 37°C in a humidified atmosphere containing 5% CO_2_. Primary human epithelial melanocytes (HEMs, newborn/darkly pigmented skin) were obtained from Thermo Fisher Scientific. The melanocyte basal medium consisted of Medium 254 with human melanocyte growth supplement (Thermo Fisher Scientific). The medium was changed once every other day for culture maintenance. Cells were not used beyond passage 10. A three-dimensional human skin model (3D-HSM, MEL-300-B) was cultured at 37°C with 5% CO_2_ in EPI-100LLMM medium (MatTek Corporation, Ashland, MA, USA). All human-derived cells used were derived and established by accredited institutions of Thermo Fisher Scientific and MatTek Corporation. Cells obtained through these institutions comply with the applicable legal and ethical guidelines of the United States. In all cases, consent for use of the cells for research purposes was obtained by these institutions from the donor or the donor's legal next of kin.

### Melanin quantification

B16F10 cells were seeded in 100-mm dishes at a density of 1.0 × 10^5^ cells per dish and then cultured in DMEM supplemented with 10% FBS at 37°C with 5% CO_2_ until they reached 80–90% confluence. After replacing the culture medium with fresh medium supplemented with 2% FBS, cells were coincubated with the extracts or test compounds for 24 h. HEMs were seeded in 100-mm dishes at a density of 1.0 × 10^5^ cells per dish and then incubated in Medium 254 with human melanocyte growth supplement for 24 h. After replacing the culture medium with fresh medium, cells were coincubated with the test compounds for the indicated lengths of time. The culture medium with or without the test compounds was changed every other day. All extracts and test compounds were first diluted in culture media at 37°C before incubation with the cells. In the 3D-HSM experiment, the skin equivalents were preincubated in EPI-100LLMM medium at 37°C with 5% CO_2_ overnight. After replacing the culture medium with fresh medium, the 3D-HSM was coincubated with the test compounds for 14 days. The compounds were first diluted in 75 mM Tris-HCl (pH 8.8) at room temperature. Next, 0.1 ml of buffered solution without (control) or with each compound was applied to the stratum corneum side. The medium and each buffered solution was changed every other day. Melanin content in cells and 3D-HSM was measured by modifying previously reported methods [12, 13]. Cells or 3D-HSM were washed three times with PBS. For the spectrophotometric calculation of total melanin, the pellets from cells or 3D-HSM were washed with 5% trichloroacetate, ethanol–ether (3:1 v/v), and ether. Next, the pellets were dried at 50°C for 1 h. Dried precipitates were solubilized with 2 M sodium hydroxide or SOLVABLE^TM^ (Perkin-Elmer, Waltham, MA, USA) by heating at 80°C for 30 min. Lysates were clarified by centrifugation, and the supernatants were transferred to a clean tube. Each sample was monitored at 405 nm using an 1420 ARVO series multilabel counter (Perkin-Elmer). The estimated pigment values from cells and 3D-HSM were normalized by cell number and absorbance value in the methylthiazole tetrazolium (MTT) assay, respectively. The number of viable cells was assessed by a trypan blue exclusion assay.

### MTT assay

Cell viability was evaluated using the MTT assay kit (MatTek Corporation) per the manufacturer’s instructions with slight modifications. The 3D-HSM was placed in a 24-well plate, 0.3 ml of MTT solution (1 mg/ml) was added to each well, and the tissue was incubated at 37°C in 5% CO_2_ for 3 h. After incubation, the tissue was washed three times with PBS. For the MTT extraction, 2 ml of a 0.04 N HCl solution in isopropanol was added to each well, and the plate was gently shaken at room temperature for 2 h. The absorbance of the extracts was measured at 570 nm using a 1420 ARVO series multilabel counter.

### HPLC

For HPLC, we employed a gradient system equipped with a syringe-loading sample injector (Model 7125, Rheodyne, Cotati, CA, USA), dual pumps, and a multi-UV detector (Model PU-2089 and MD-2010, respectively, JASCO, Tokyo, Japan). Two mobile phase solvents were used. Solvent A was prepared by adding concentrated formic acid (0.1%) to deionized water. Solvent B was HPLC-grade acetonitrile. The HPLC method consisted of a reverse-phase column (Develosil C_18_, 5 μm, 4.6 × 250 mm, Nomura Chemical, Aichi, Japan) and the following mobile phase gradient: 0–40 min with a linear gradient from 95% solvent A to 100% solvent B and 40–50 min hold 100% of solvent B. The flow rate was constant at 1.0 ml/min.

### Determination of total phenolic compounds

The concentration of total phenolic compounds in each extract was evaluated spectrophotometrically using Folin–Ciocalteu reagent and a previously published method with slight modifications [14]. Briefly, 0.1 ml of each sample or standard previously dissolved in ethanol was diluted with water to a volume of 0.45 ml. Next, 0.5 ml of Folin–Ciocalteu phenol reagent was added, and the test tubes were vigorously agitated. 0.4 ml of a 1 M sodium carbonate solution was subsequently added, and the tubes were thoroughly agitated again. The mixtures were incubated at room temperature and protected from light for 1 h. The absorbance of the resulting reaction mixtures was measured at 750 nm with an Ultrospec 4300 pro UV/visible spectrophotometer (GE Healthcare). The concentration of total phenolic compounds for each extract was calculated based on the standard curve obtained using gallic acid.

### Determination of total flavonoids

The quantification of total flavonoids in each extract was completed using the Lamaison and Carnat method modified for a microplate assay [15]. Each 100-μl sample was added to a 96-well plate, followed by addition of 100 μl of a 2% AlCl_3_ solution in MeOH. After 10 min, the absorbance at 415 nm was measured with a 1420 ARVO series multilabel counter. A standard curve was developed using quercetin.

### Quantitative PCR (qPCR)

Total RNA was isolated from cultured cells using an RNeasy Mini Kit (Qiagen, Mississauga, Canada) per the manufacturer’s instructions. RNA was stored in RNase-free water at −80°C prior to reverse transcription. First-strand cDNA was synthesized with 1 μg of total RNA using a PrimeScript II 1st strand cDNA Synthesis Kit (Takara Bio, Shiga, Japan) per the manufacturer’s instructions. mRNA expression levels of target genes were measured using an Applied Biosystems 7500 Real Time PCR System (Applied Biosystems, Foster City, CA, USA) and the following TaqMan Gene Expression Assays: *Mitf* (assay ID Mm00434954_m1); *Mlph* (assay ID Mm01286059_m1); and *Gapdh* (assay ID Mm99999915_g1). All reactions were performed in triplicate. *Gapdh* was used as a housekeeping gene for normalization. The relative amount of mRNA was calculated using the comparative C_T_ method.

### Immunoblotting

B16F10 cells were lysed using a lysis buffer (150 mM NaCl; 50 mM Tris, pH 8.0; 1% NP-40; 0.5% sodium deoxycholate; and protease inhibitors). Each extract was subjected to SDS-PAGE, and proteins were transferred to polyvinylidene fluoride membranes using a semi-dry blotter (Bio-Rad Laboratories, Hercules, CA, USA). After the transfer, the membranes were incubated in a blocking solution [0.3–1% dried skimmed milk in Tris-buffered saline containing 0.1% Tween-20 (TBS-T)] for 1 h to reduce nonspecific binding. Next, membranes were incubated with primary antibodies overnight at 4°C. The blots were washed and incubated with HRP-conjugated secondary antibodies for 1 h. Proteins were detected using ImmunoStar LD (Wako Pure Chemical).

### Immunofluorescence and melanosome distribution assay

B16F10 cells or HEMs on collagen coated coverslips were fixed for 15 min in 4% paraformaldehyde, permeabilized with 0.1% Triton-X 100 for 10 min, and incubated in a blocking solution (3% BSA in PBS) for 1 h. To detect actin, cells were treated with diluted Alexa Fluor 594 phalloidin for 20 min. For TYRP1 coimmunostaining, cells were exposed to an anti-TYRP1 antibody (1:75 dilution) in PBS containing 1% BSA for 16 h at 4°C and subsequently incubated with an Alexa Fluor 488-conjugated anti-goat antibody (1:400 dilution) for 1 h. For nuclear counterstaining, cells were treated with 1 μg/ml DAPI. Mounted samples were observed using a TCS SP2 AOBS confocal laser microscope (Leica Microsystems, Wetzlar, Germany). Melanosome distribution assays were performed using a previously published method with slight modifications (more than 100 cells/cover glass, three independent glasses for each sample) [16]. Cell images were randomly acquired, and cells with more than 50% of melanosomes around the nucleus were labeled as aggregated.

### Statistical analyses

The data are expressed as the mean ± SD from at least three independent experiments. Statistical analyses were performed using the Tukey–Kramer test. Asterisks (*p < 0.05 and **p < 0.01) and daggers (^††^*p* < 0.01) indicate statistical significance when compared with the control group and the indicated group, respectively.

## Results

**Fig 1:**
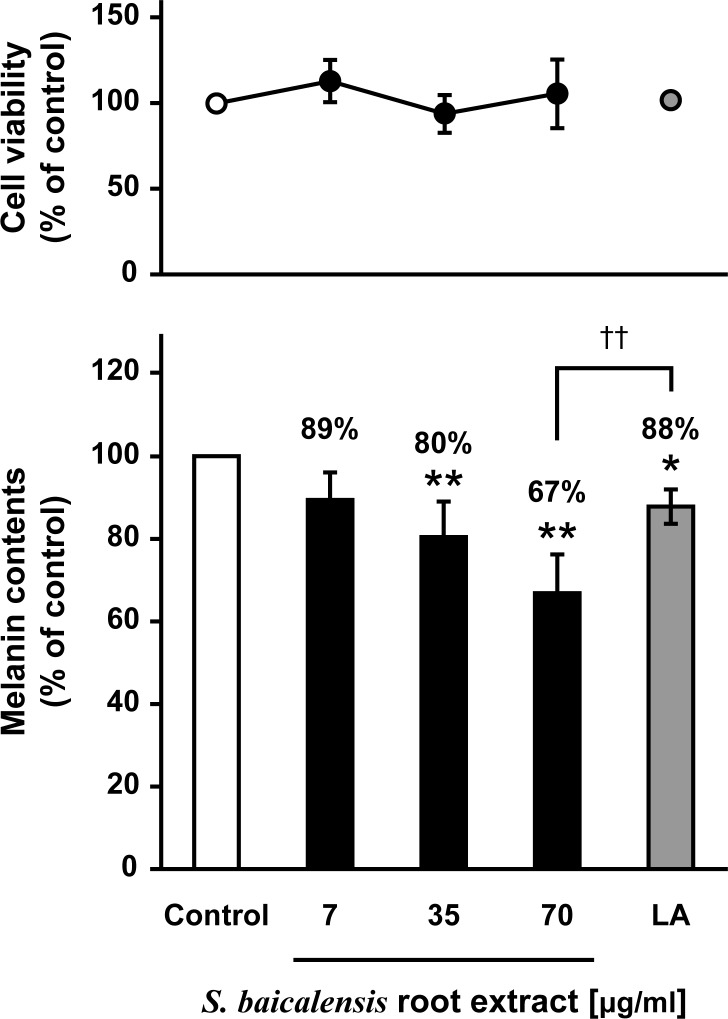
*S*. *baicalensis* root extract inhibits melanogenesis in B16F10 melanoma cells. B16F10 cells were cultured for 24 h with LA (100 μM) or the indicated concentrations of *S*. *baicalensis* root extracted with 70% ethanol.

**Fig 2:**
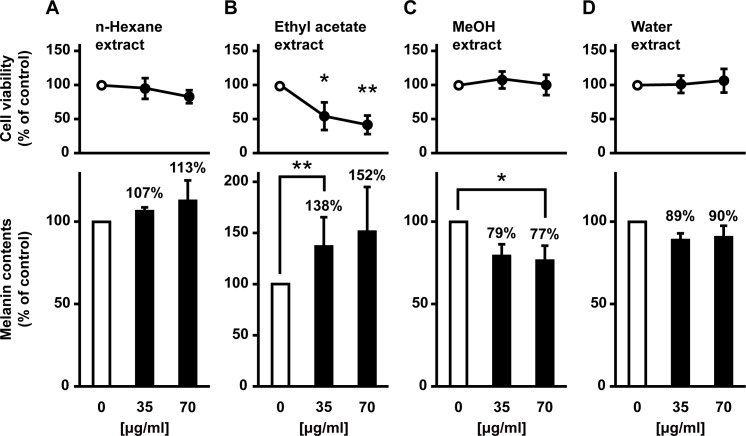
Inhibitory effects of four different *S*. *baicalensis* root extracts on melanogenesis in B16F10 melanoma cells. B16F10 cells were cultured for 24 h after the addition of *S*. *baicalensis* root extracted with different solvents: *n*-hexane (**A**), ethyl acetate (**B**), MeOH (**C**), and water (**D**).

To examine the depigmenting ability of *S*. *baicalensis*, we first quantified melanin levels using cultured mouse B16 melanoma cells. As shown in Fig 1, the *S*. *baicalensis* extract significantly decreased melanin levels in a dose-dependent manner between 35 and 70 μg/ml. At a concentration of 70 μg/ml, the *S*. *baicalensis* extract inhibited melanin formation more effectively than 100 μM of LA, which was used as a positive control [17]. To determine the most efficient extraction of *S*. *baicalensis*, we examined the inhibitory activity of each extract generated from four solvents: *n*-hexane, ethyl acetate, MeOH, and water. After the treatment of B16F10 cells with each extract for 24 h, the melanin content was assessed. The *S*. *baicalensis* extract eluted by MeOH resulted in a significant decrease in melanin content (Fig 2C), whereas no decrease was observed after treatment with the other three extracts (Fig 2A, 2B and 2D). In fact, the extract eluted by ethyl acetate tended to increase melanin content and produced toxicity (Fig 2B). These results suggest that *S*. *baicalensis* is capable of inhibiting melanogenesis and its active components can be efficiently extracted by alcohols such as MeOH.

**Fig 3:**
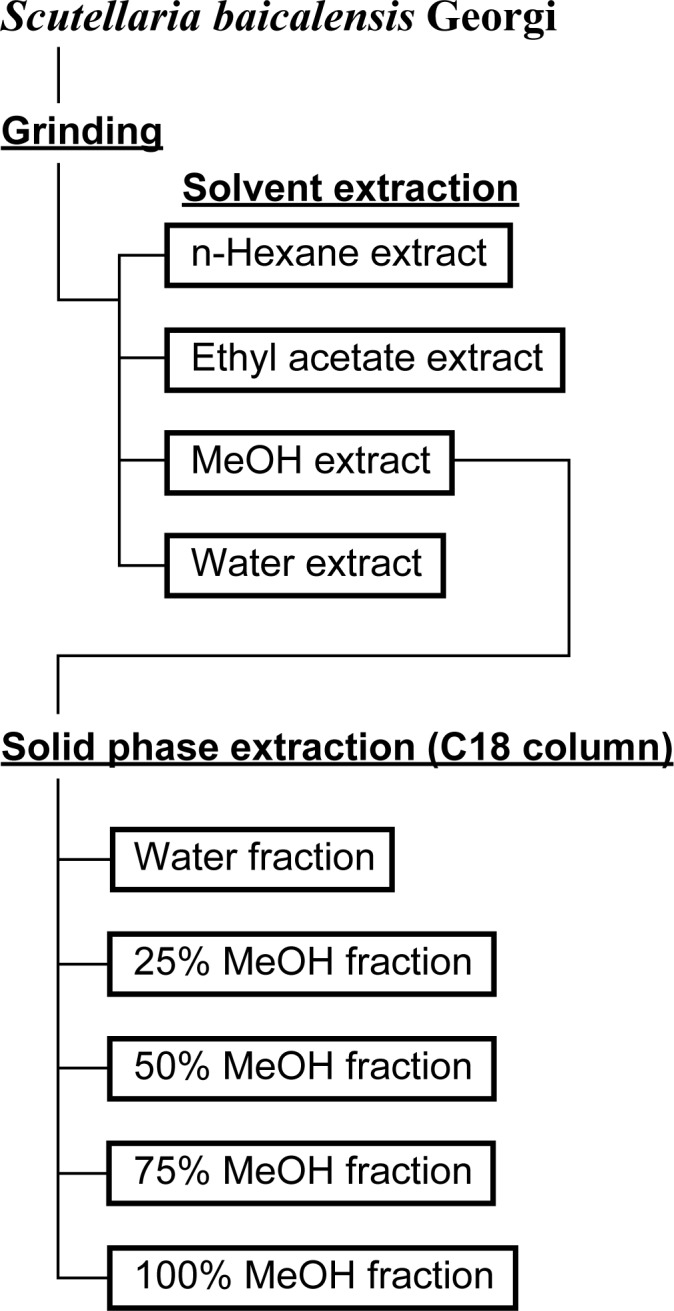
Procedure for fractionation of *S*. *baicalensis* root.

**Fig 4:**
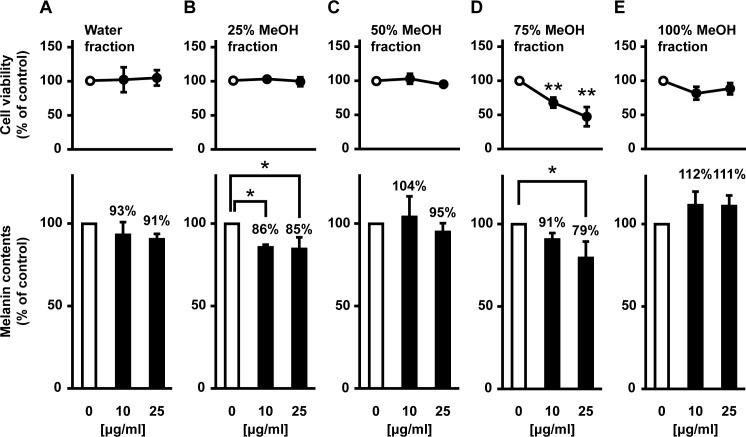
Inhibitory effects of the MeOH fractions of *S*. *baicalensis* root on melanogenesis in B16F10 melanoma cells. B16F10 cells were cultured for 24 h after the addition of one of the following five fractions: water (**A**), 25% (**B**), 50% (**C**), 75% (**D**), and 100% MeOH (**E**).

**Fig 5:**
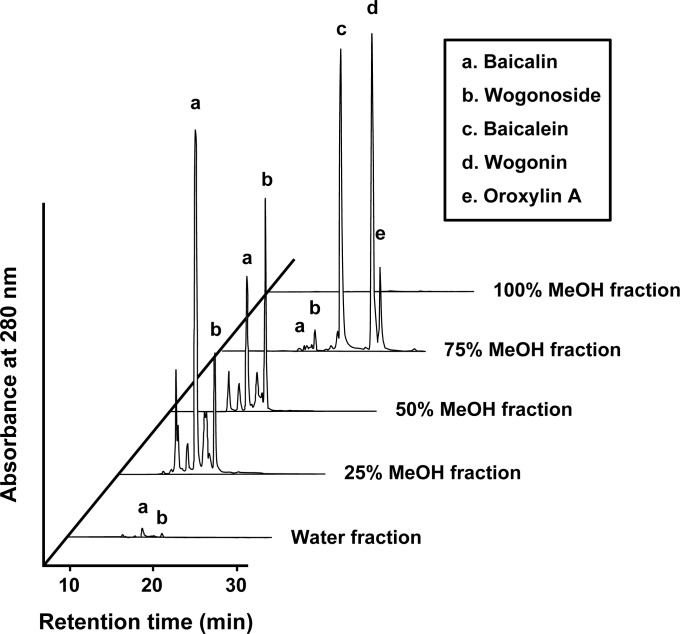
HPLC chromatogram of *S*. *baicalensis* root extracted with MeOH. HPLC profiles for each of the fractions were obtained using a C18 column (i.d. 4.6 × 250 mm).

To identify the active components that inhibit melanin synthesis, the *S*. *baicalensis* MeOH extract was sequentially fractionated into five samples using a C18 solid-phase extraction column (Fig 3). We assessed the effect of these five fractions on melanogenesis. As shown in Fig 4, only the 25% MeOH and 75% MeOH fractions decreased melanin content. The 75% MeOH fraction also exhibited significant cytotoxicity against B16F10 cells. Quantitative assays revealed that the total phenolic compounds and flavonoids were abundant in the following order: 75%, 25%, and 50% MeOH fractions (Table 1). Based on these results, we speculated that certain flavonoids in *S*. *baicalensis* were responsible for the inhibition of melanogenesis. Previous reports identified the major flavonoids found in *S*. *baicalensis* (S1 Fig) [18]. Using an HPLC-diode array detector and liquid chromatography-tandem mass spectrometry, we compared the corresponding retention times and UV and mass spectra of samples with the standards indicated in the literature. We identified the five characteristic peaks as baicalin (peak a), wogonoside (peak b), baicalein (peak c), wogonin (peak d), and oroxylin A (peak e) (Fig 5). The assignment of each peak revealed that the fractions eluted with 25% and 50% MeOH contained abundant flavone glycosides, particularly baicalin and wogonoside (Table 2). The main components present in the 75% MeOH fraction were three aglycone flavones: baicalein, wogonin, and oroxylin A. In contrast, both the water and 100% MeOH fractions contained almost no constituents.

**Table 1:** Fraction yields, total phenolic compounds, and flavonoids of the five fractions obtained from the MeOH extract of *S*. *baicalensis* root.

	Extract yield (%)	Total phenolic compounds (%)	Flavonoids (%)
**Water fraction**	79.73	3.80 ± 0.00	0.37 ± 0.08
**25% MeOH fraction**	3.65	21.66 ± 0.04	18.60 ± 0.59
**50% MeOH fraction**	9.99	14.91 ± 0.04	14.15 ± 0.21
**75% MeOH fraction**	4.12	53.54 ± 0.01	63.50 ± 1.14
**100% MeOH fraction**	2.51	6.09 ± 0.01	3.38 ± 0.23
**Total**	100.00	100.00	100.00

**Table 2:** The constituent concentrations of the five MeOH *S*. *baicalensis* root fractions.

	Baicalin (μM)	Wogonoside (μM)	Baicalein (μM)	Wogonin (μM)	Oroxylin A (μM)
**Water fraction**	1.2	0.2	0.0	0.0	0.0
**25% MeOH fraction**	18.9	5.1	0.0	0.0	0.0
**50% MeOH fraction**	5.0	6.8	0.0	0.0	0.0
**75% MeOH fraction**	0.0	0.0	22.6	19.0	6.0
**100% MeOH fraction**	0.0	0.0	0.6	0.2	0.2

Each fraction was analyzed at a concentration of 25 μg/ml.

**Fig 6:**
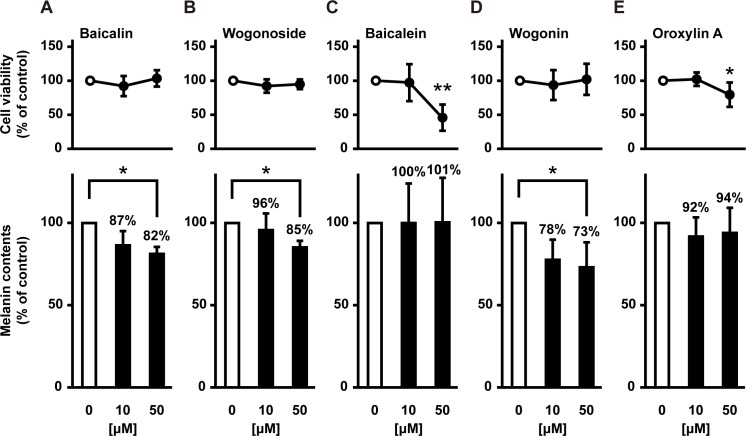
Inhibitory effect of *S*. *baicalensis* root constituents on melanogenesis in B16F10 melanoma cells. B16F10 cells were cultured for 24 h with one of the five major flavones of *S*. *baicalensis*: baicalin (**A**), wogonoside (**B**), baicalein (**C**), wogonin (**D**), and oroxylin A (**E**).

We assessed the effect of five major flavones: baicalin, wogonoside, baicalein, wogonin, and oroxylin A. As shown in Fig 6, 50 μM of baicalin, wogonoside, and wogonin significantly inhibited melanin synthesis without causing cytotoxicity, whereas baicalein and oroxylin A did not exert this effect. Wogonin strongly inhibited and decreased melanin content by 26.7% when compared with the control. In contrast, baicalein and oroxylin A caused marked cytotoxicity in the absence of any inhibitory effects on melanogenesis. These results suggest that two glycosides (i.e., baicalin and wogonoside) and wogonin are the active components responsible for the antimelanogenic effects of the 25% MeOH and 75% MeOH fractions, respectively. The 75% MeOH fraction also contained baicalein and oroxylin A (Table 2), which likely contribute to high cytotoxicity of this fraction. Thus, baicalin, wogonoside, and wogonin are the active constituents of *S*. *baicalensis* that inhibit melanin formation in B16F10 cells.

**Fig 7:**
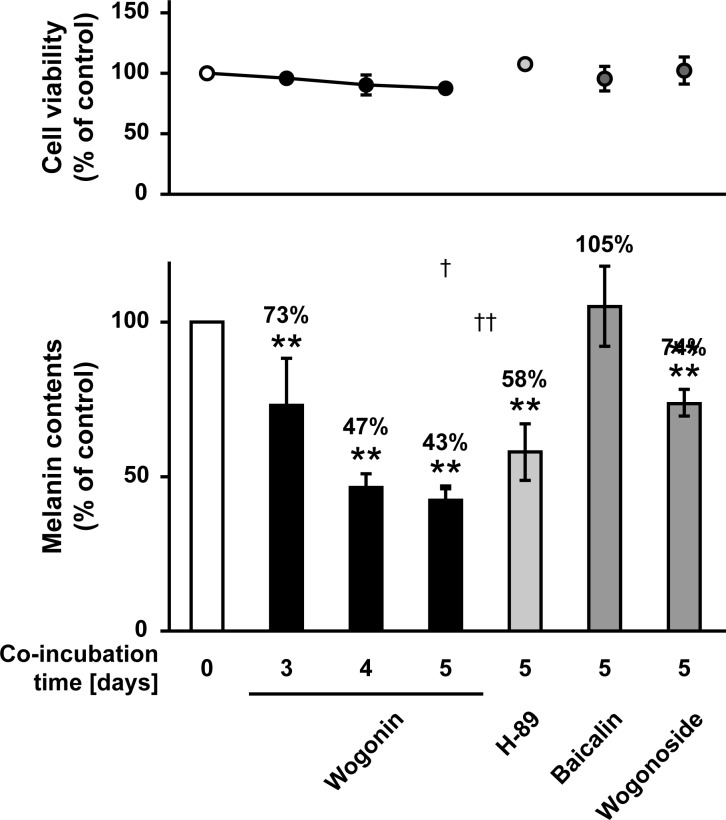
The inhibitory effect of three *S*. *baicalensis* root constituents on melanogenesis in HEMs. HEMs were cultured for the indicated number of days with 25 μM wogonin, baicalin, and wogonoside or 1 μM H-89.

**Fig 8:**
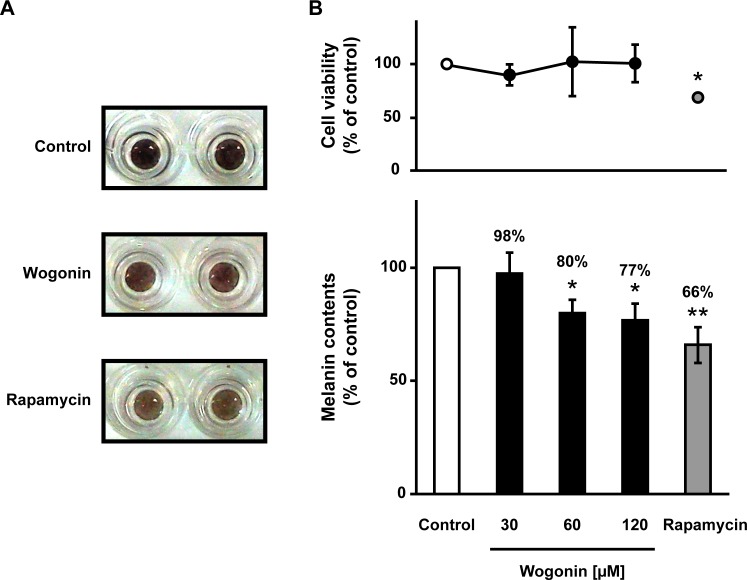
The inhibitory effect of wogonin on melanogenesis in a 3D-HSM. The 3D-HSM was cultured for 14 days with 1 μM rapamycin or wogonin at the indicated concentrations. (**A**) The photographs of 3D-HSM were taken after 14 days in culture with 1 μM rapamycin or 120 μM wogonin. (**B**) The level of melanogenesis was determined after 14 days in culture.

To elucidate the effect of the three active components of *S*. *baicalensis* root under normal cell conditions, we examined melanin levels in HEMs after exposure to each compound. As shown in Fig 7, exposure to wogonin at a concentration of 25 μM for 3–5 days inhibited melanin formation in a time-dependent manner without affecting the growth or survival of HEMs. Exposure to wogonin for 4–5 days showed a tendency of stronger inhibition when compared with H-89, which blocks melanin synthesis via cyclic AMP-dependent protein kinase-specific inhibition [19]. In contrast, baicalin and wogonoside had no effect or a weak effect on melanogenesis in HEMs (Fig 7). We also assessed the activity of wogonin using a 3D-HSM. As shown in Fig 8, exposure of the 3D-HSM to wogonin at concentrations of 30–120 μM did not cause cytotoxicity. Wogonin markedly lightened the color of the 3D-HSM and decreased melanin content by up to 23.0% when compared with the control (Fig 8A and 8B). Rapamycin, which lightens skin color via autophagy [13], decreased melanin content by 34.0% but also caused cytotoxicity. These results suggest that among the three active components identified in the B16F10 model, wogonin and wogonoside consistently downregulate melanin formation in both melanoma and normal cell assays.

**Fig 9:**
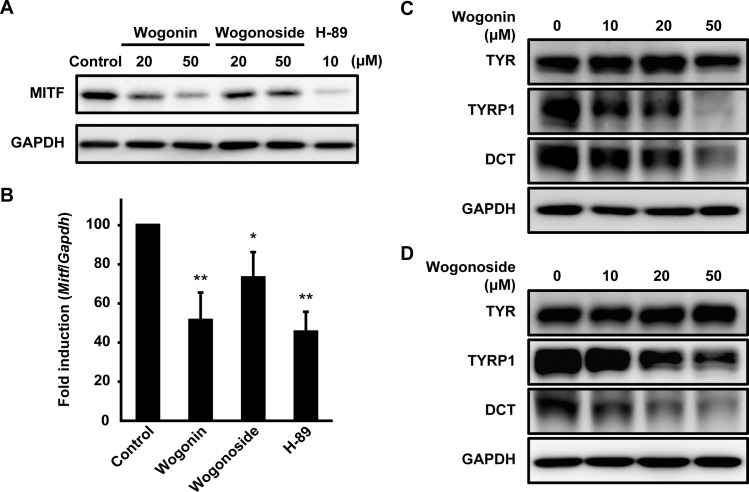
Wogonin and its glycoside downregulate the expression of melanogenesis-related proteins by suppressing MITF expression. (**A**) After the treatment of B16F10 cells with wogonin, wogonoside, or H-89 for 24 h, MITF protein expression was determined by immunoblotting with an anti-MITF antibody. (**B**) After 24 h of exposure to 50 μM wogonin, wogonoside, or 10 μM H-89, *Mitf* mRNA expression was quantified using qPCR. (**C–D**) B16F10 cells were cultured for 3 days with wogonin or wogonoside at the indicated concentration. The expression of each protein was analyzed using immunoblotting with specific antibodies for TYR, TYRP1, and DCT.

Wogonin has been used as a treatment for cancers and ocular diseases [9, 10]. In these previous studies, wogonin exhibited antitumor effects by inhibiting the extracellular regulated protein kinase and Wnt/beta-catenin pathways. In melanocytes, these pathways play a key role in MITF expression [20, 21]. We found that wogonin and wogonoside significantly reduced MITF expression in B16F10 cells (Fig 9A and 9B). In addition, wogonin and wogonoside significantly inhibited TYRP1 and DCT but not TYR (Fig 9C and 9D). These results suggest that wogonin and its glycoside downregulate the expression of melanogenesis-related proteins by suppressing MITF expression.

**Fig 10:**
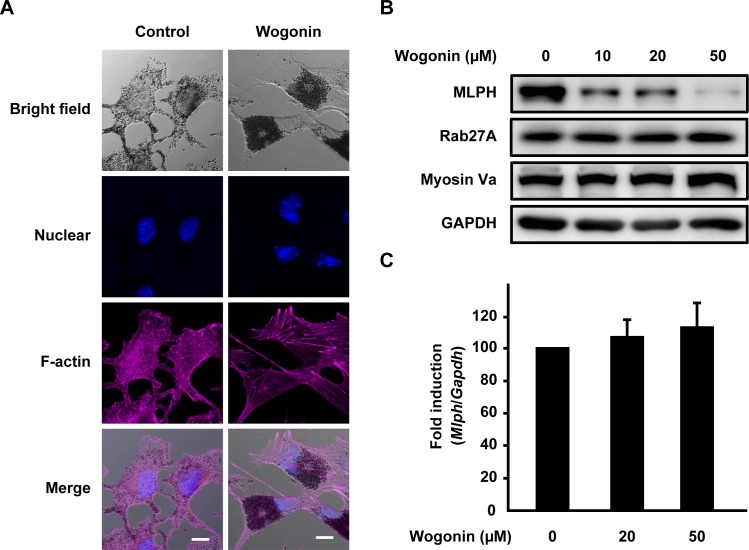
Wogonin induces the perinuclear aggregation of melanosomes in B16F10 cells. B16F10 cells were cultured for 3 days with wogonin at a concentration of 50 μM (**A**) or the indicated concentrations (**B–C**). (**A**) Nuclei (blue) and F-actin (magenta) were analyzed by confocal microscopy. Bright field images show the melanosome (black pigment) distribution. Scale bar = 10 μm. (**B**) MLPH, Rab27A, and myosin Va levels were determined by immunoblotting with anti-MLPH, Rab27A, and myosin Va antibodies, respectively. (**C**) The expression of *Mlph* mRNA was quantified using qPCR.

**Fig 11:**
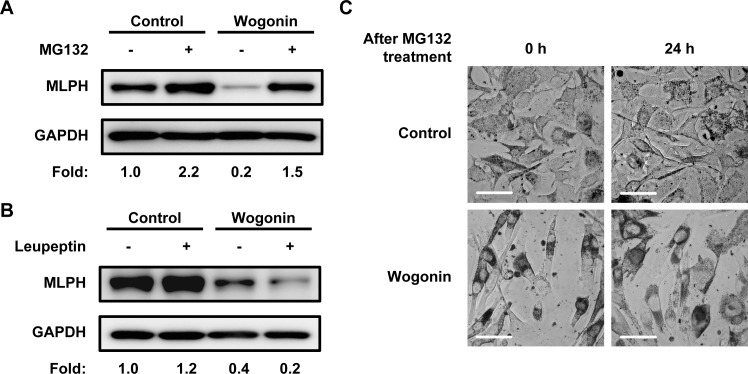
Wogonin induces MLPH degradation via the calpain/proteasomal pathway. B16F10 cells were cultured for 2 days with or without 50 μM wogonin. Following replacement of the medium, B16F10 cells were cultured for an additional 24 h with or without wogonin (50 μM) in combination with MG132 (120 nM) or leupeptin (20 μM). (**A–B**) MLPH levels were determined by immunoblotting with an anti-MLPH antibody. (**C**) Bright field images show the melanosome distribution. Scale bar = 50 μm.

**Fig 12:**
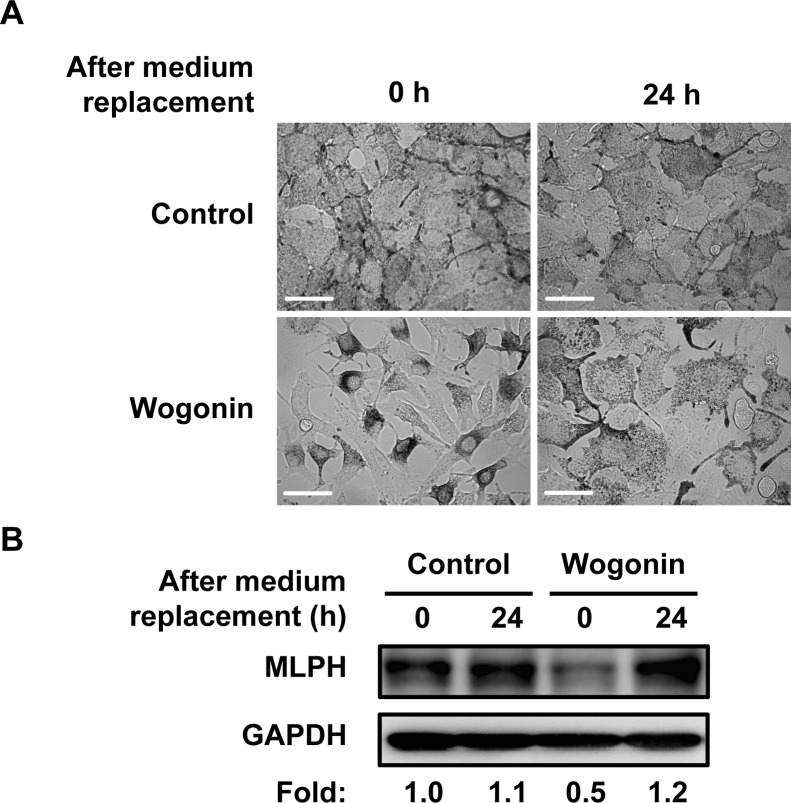
Cessation of treatment with wogonin reverses melanosome aggregation and the reduction in MLPH. B16F10 cells were cultured for 3 days with or without 50 μM wogonin. Following replacement of wogonin-free medium, B16F10 cells were cultured for an additional 24 h. (**A**) Bright field images show the melanosome distribution. Scale bar = 50 μm. (**B**) The MLPH level was determined by immunoblotting with an anti-MLPH antibody.

**Fig 13:**
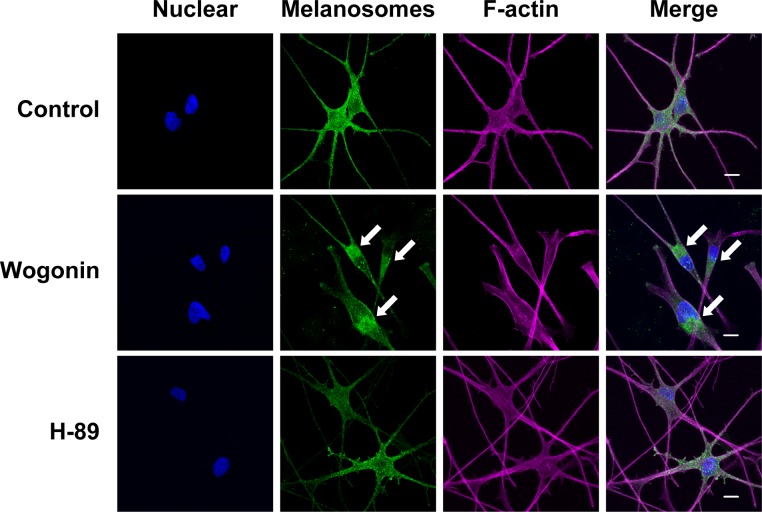
Wogonin induces perinuclear aggregation of melanosomes in HEMs. HEMs were cultured for 3 days with 25 μM wogonin or 1 μM H-89. Melanosomes were immunostained with an anti-TYRP1 antibody and visualized with an Alexa Fluor 488 secondary antibody. Nuclei and F-actin were stained with DAPI and phalloidin with a fluorescent analog (Alexa Fluor 594), respectively. Nuclei and F-actin fluorescence and immunostaining for melanosomes were analyzed by confocal microscopy. Scale bar = 10 μm. The arrows indicate perinuclear aggregation.

In culture, we found that melanosomes in wogonin-treated B16F10 cells were localized to the perinuclear region and conspicuously absent from the cell periphery when compared with the whole-cell distribution pattern of the control (Fig 10A). Melanosomes in mammalian melanocytes are intracellularly transported along microtubules and actin filaments [4]. Actin-based melanosome transport is mediated by Rab27A, myosin Va, and MLPH. Any depletion or defect in these proteins disrupts traffic and induces abnormal melanosome distribution [6–8]. The perinuclear melanosome aggregation induced by wogonin exhibited similarities to the distribution patterns induced by myosin Va, Rab27A, and MLPH deficiencies [7, 8, 22]. Therefore, we investigated whether wogonin downregulates actin-based melanosome transport-related molecules (e.g., Rab27A) in B16F10 cells. Wogonin caused a dose-dependent reduction in MLPH protein (Fig 10B), whereas the protein levels of Rab27A and myosin Va were not affected by wogonin treatment. The *Mlph* mRNA level was not affected by wogonin (Fig 10C), suggesting that wogonin had little inhibitory effect on the gene transcription of *Mlph*. MLPH contains multiple PEST-like sequences that are highly sensitive to proteolysis [23]. To determine whether wogonin promotes MLPH degradation, we examined the effects of the calpain/proteasome inhibitor MG132 and the lysosome inhibitor leupeptin on the wogonin-induced decrease in MLPH protein. MG132 but not leupeptin effectively blocked the reduction in MLPH (Fig 11A and 11B). These results suggest that wogonin promotes the proteolytic degradation of MLPH via the calpain/proteasomal pathway. In addition, the treatment of cells with MG132 failed to rescue the change in melanosome distribution induced by wogonin (Fig 11C), suggesting that wogonin induces MLPH dysfunction. Upon removal of wogonin from the culture medium, the perinuclear aggregation was reversed (Fig 12A) and MLPH levels were restored (Fig 12B), indicating that the inhibitory effect of wogonin on both melanosome transport and MLPH protein expression only occurs in the presence of wogonin. These results suggest that MLPH turnover following removal of wogonin normalizes intracellular melanosome transport. Melanosome localization in HEMs was assessed by immunostaining for TYRP1 as a melanosome marker [24]. Pronounced perinuclear localization of melanosomes was observed in HEMs after treatment with wogonin but not H-89 when compared with control cells (Fig 13). These results collectively suggest that wogonin downregulates constitutive actin-based melanosome transport by accelerating MLPH degradation.

**Fig 14:**
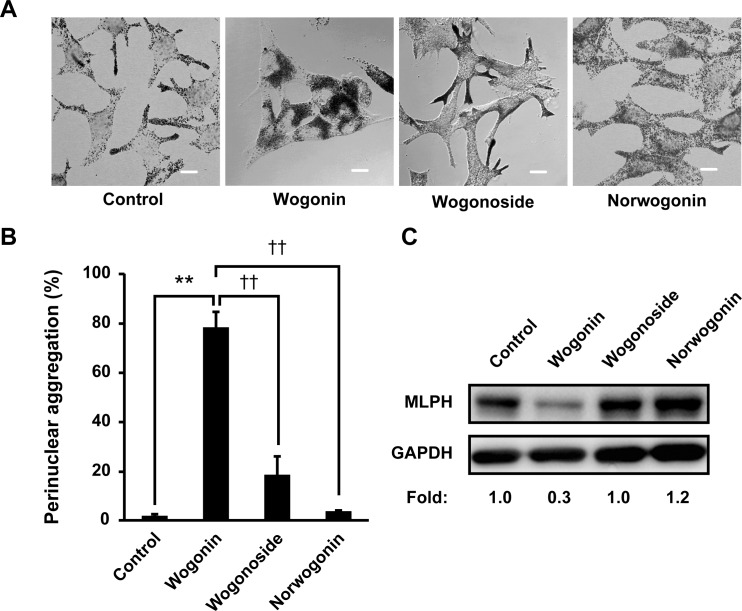
Glycosidation or 8-hydroxylation of wogonin impairs its inhibitory effect on melanosome transport. B16F10 cells were cultured for 3 days with 50 μM wogonin, wogonoside, or norwogonin. (**A**) Bright field images show the melanosome distribution. Scale bar = 10 μm. (**B**) The results are expressed as the percentage of cells showing perinuclear melanosome aggregation. (**C**) MLPH levels were determined by immunoblotting with an anti-MLPH antibody.

**Fig 15:**
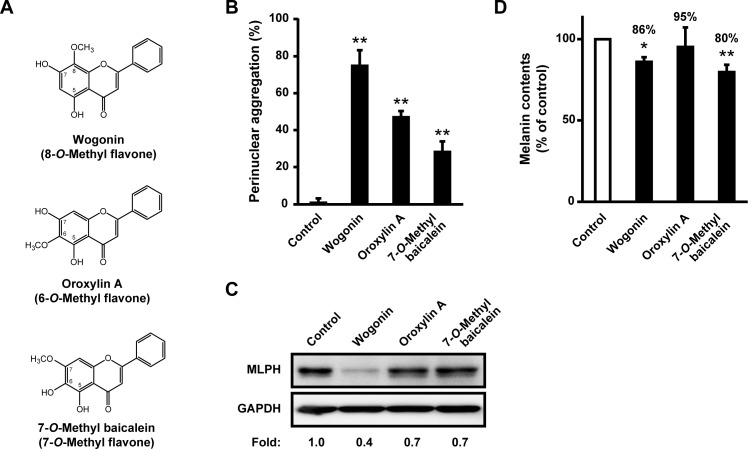
An *O*-methyl group in the flavone A-ring plays a key role in downregulating melanosome transport. (**A**) The structures of three mono-*O*-methylated flavones. (**B–C**) B16F10 cells were cultured for 3 days with 50 μM wogonin or each of the two wogonin analogs. The results are expressed as the percentage of cells showing perinuclear melanosome aggregation. MLPH levels were determined by immunoblotting with an anti-MLPH antibody. (**D**) B16F10 cells were cultured for 24 h with 50 μM of each flavone. The melanin content in each culture was quantified.

To determine the structural aspects of wogonin responsible for melanosome transport defects, B16F10 cells were cultured with several wogonin analogs. As shown in Fig 14A and 14B, norwogonin, an 8-hydroxylated wogonin, completely failed to aggregate intracellular melanosomes when compared with the perinuclear aggregation mediated by wogonin. This result indicates that the hydroxyl substitution of the 8-*O*-methyl group in wogonin caused a loss of function. In addition, wogonoside, a glycoside of wogonin, showed a noticeably weaker effect on melanosome localization when compared with wogonin. This result suggests that the glycoside conjugation of wogonin inhibited its ability to regulate melanosome transport. Next, we assessed the effect of these structural features on MLPH levels. As shown in Fig 14C, B16F10 cells cultured with wogonoside and norwogonin showed almost no detectable reduction in the protein level of MLPH when compared with wogonin-treated melanoma cells. Therefore, we hypothesized that the *O*-methyl group in the flavone A-ring may disturb melanosome transport. In support of our hypothesis, the 7-*O*-methylation of baicalein led to an acquired ability to impair melanosome transport (S2 Fig). To define the superiority of the *O*-methyl position on the flavone A-ring on melanosome transport, a comparative study of three structural isomers, i.e., wogonin, oroxylin A, and 7-*O*-methyl baicalein, was performed (Fig 15A). Oroxylin A, a 6-*O*-methyl-positioned isomer, inhibited melanosome dispersion but to a lesser extent (47.5%) than wogonin (77.9%; Fig 15B). Among these three flavones, wogonin had the strongest inhibitory effect at 77.9%, whereas 7-*O*-methyl baicalein had the weakest inhibitory effect at 28.6%. These results indicate that 8-*O*-methyl flavones, including wogonin, are the most potent melanosome transport inhibitors. *O*-Methyl-positioned flavones also showed inhibitory effects on MLPH levels (Fig 15C), suggesting that inhibition of melanosome transport by *O*-methylated flavones was due to the downregulation of MLPH. In 6-, 8-, and 7-*O*-methylated flavones, the extent of antimelanogenic effectiveness decreased, respectively (Fig 15D), indicating a lack of concordance between their effectiveness against melanogenesis and intracellular melanosome transport. These results suggest that the 8-*O*-methyl group of wogonin obligately downregulated melanosome transport.

## Discussion

This study reports three major findings: (1) *S*. *baicalensis* potently inhibits melanogenesis, (2) wogonin and wogonoside are the active components of *S*. *baicalensis*, and (3) the *O*-methylated flavones in *S*. *baicalensis*, such as wogonin, inhibit intracellular melanosome transport. The antimelanogenic action of wogonin and its glucoside are likely due to the inhibition of MITF expression (Fig 9). In this study, B16F10 cells were cultured in DMEM without additional stimulation, but recent reports have shown that highly concentrated _L_-tyrosine in DMEM exerts a hormone-like action and stimulates melanogenesis [25, 26]. Therefore, we also evaluated the effect of these two flavones on melanogenesis in RPMI 1640 medium (RPMI), which contains a lower concentration of _L_-tyrosine than DMEM. The two flavones significantly decreased the melanin content of B16F10 cells cultured in _L_-tyrosine-supplemented RPMI (S3 Fig), whereas no decrease was observed in cells cultured in RPMI. This result suggests that the flavones did not exert an inhibitory effect on melanogenesis in a ground state. We showed that wogonin and wogonoside downregulate TYRP1 and DCT, but preserve TYR (Fig 9). This change may shift eumelanogenesis towards the 5,6-dihydroxyindole oxidation sub-pathway [27]. A previous report showed that TYRP1 deficiency produced light brown pigmentation in culture [28]. To detect changes in color other than discoloration, we assessed the hue of melanin produced by melanocytes from moderately pigmented skin. The color of melanin from wogonin-treated cells were light brown and clearly different from the black to dark brown color of control cells (S4 Fig). However, the discoloration caused by H-89 treatment also resulted in a visibly pale brown; thus, it is difficult to determine which characteristics are specific to the 5,6-dihydroxyindole sub-pathway. Recently, sex-determining region Y-box 9 (SOX9) and SOX10 were identified as key regulators of the expression of melanogenesis-related genes, including the gene encoding MITF [29, 30]. Wogonin severely downregulated SOX9 and moderately upregulated SOX10 (S5 Fig), indicating that SOX9 is a potential target for the effect of these *S*. *baicalensis* flavones.

We focused on melanosome transport-related proteins serving as actin-based transport and found a decline in the protein level of MLPH induced by wogonin (Fig 10B). The calpain/proteasome inhibitor MG132 attenuated the MLPH reduction induced by wogonin (Fig 11A). Wogonin-mediated adaptor dysfunction was postulated to induce the early intervention of proteases, thus leading to MLPH degradation. Recent patent applications reported that several plant extracts, including *Sesamum indicum* L. and *Cassia mimosoides* L., suppressed the levels of multiple melanosome transport-related proteins in HEMs and resulted in melanosome transport disorders [31, 32]. In contrast, the inhibitory effect of wogonin appeared to be specific to MLPH. Several recent reports showed that MITF serves as a transcription factor for Rab27A but not MLPH [33, 34]. In this study, wogonin downregulated the protein levels of MLPH but not Rab27A or myosin Va (Fig 10B). This result suggests that the moderate decrease in MITF induced by wogonin has little or no influence on the expression of actin-based transport molecules, such as Rab27A. MITF also controls the expression of other loci related to melanosome transport, including Ocular Albinism type 1 (OA1) [30, 34–35], which serves as a microtubule-based motor molecule. Interestingly, wogonin reduced the expression of OA1 mRNA (S6 Fig), indicating that wogonin affects both the microtubule-based and actin-based transport of melanosomes. Palmisano et al. reported that OA1 deficiency leads to the abnormal distribution of melanosomes towards the cell periphery, which appears opposite to the melanosome distribution due to dysfunction of actin-mediated transport [35]. According to this previous study, the characteristic disturbance of melanosome by OA1 deficiency was rescued by the inhibition of actin-based transport. We did not observe the peripheral accumulation of melanosomes in wogonin-treated cells. Thus, we speculate that the disorder of actin-based transport by wogonin masks the simultaneous malfunction of OA1. The reduction in melanin content induced by wogonin may be partially due to the decline in OA1 expression because OA1 deficient pigment cells were reported to show reduced melanin content and macromelanosomes [35].

MLPH is an adapter protein that links Rab27A-melanosomes to myosin Va, and a defect in MLPH produces a characteristic depigmentation [36]. Thus, the downregulation of MLPH may be helpful for correcting skin hyperpigmentation. Chang et al. reported that disrupting the MLPH-myosin Va interaction using manassantin B contributed to the inhibition of melanosome transfer from melanocytes to keratinocytes [37]. Additionally, the disruption of this interaction caused a decrease in the total melanin content in a coculture of Melan-a melanocytes and SP-1 keratinocytes. Our results raise the question of whether the wogonin-mediated loss of MLPH could be responsible for the reduction in melanin content in B16F10 cells and in melanocytes cultured alone. The siRNA-mediated knockdown of *Mlph* in B16F10 cells resulted in a moderate reduction of melanin content (S7 Fig). Combined with Fig 15, these results imply that melanosome transport defects due to the absence of MLPH slightly influenced melanogenesis.

To the best of our knowledge, our study is the first to show that the *O*-methyl group in the flavone A-ring plays a key role in the perinuclear aggregation of melanosomes. Nobiletin, an *O*-hexamethylated flavone with a 5,6,7,8-*O*-tetramethylated A-ring, failed to impair melanosome transport (S8 Fig), suggesting that the number of *O*-methylations does not yield an additive effect on melanosome transport. In addition, 5,7-*O*-dimethyl flavone, which was reported to enhance melanogenesis [38], did not show any effect on melanosome transport when compared with wogonin (S8 Fig). These flavone-treated cells appeared to be dendritic spine-shaped as compared to the star-shaped control cells. Collectively, *O*-mono-methylation, not multimethylation, of the flavone A-ring is likely essential. The bioactivities of flavones often refer to their antioxidant activities. The antioxidant capacity of flavones is generally related to the number of hydroxyl groups and available H atoms [39]. Baicalein was reported as a strong antioxidant but did not induce melanosome aggregation (S2 Fig) [40]; thus, the effect of flavones on melanosome transport is likely not linked with their antioxidant behavior. Further research is needed to assess whether hydroxyl groups in the A-ring are required for melanosome aggregation. We also showed that the effects of mono-*O*-methyl flavones on melanosome transport were strong in the following order: 8-, 6-, and 7-*O*-monomethylated (Fig 15). Thus, wogonin was the most potent inhibitor of melanosome transport among the tested flavones. Moreover, the conjugation of glycosides to wogonin caused a marked decline in the inhibitory effect on melanosome transport (Fig 14), indicating that the aglycone of wogonin may be more efficacious than the glycoside. We speculate that the aglycone was more cell permeable than the glycoside and that wogonin may act as an inhibitor against both melanogenesis and melanosome transport.

In recent years, melanization has again gained attention as a risk factor for melanoma [41]. Several studies have shown the antitumor effect of wogonin on multiple tumors, including melanomas [9, 10]. Zhao et al. used in vitro and in vivo assays to show that wogonin inhibits the cell invasion of metastatic melanoma by suppressing the PI3K/AKT and NF-κB pathways [42]. Here we showed that wogonin inhibits melanogenesis and melanosome transport by targeting MLPH in mouse melanoma B16F10 cells. Garraway et al. reported a correlation between MITF expression and poor prognosis [43]. Notably, we determined that wogonin inhibited MITF expression and melanosome transport in human melanoma MM–AN cells (S9 Fig and S1 Text). MITF not only controls melanogenic enzymes but also regulates multiple cellular processes, including differentiation, proliferation, survival, and motility [27]. Interestingly, wogonin inhibited the mRNA expression of cyclin D1 (CCND1) (S9 Fig), which regulates G1/S phase cell cycle progression and is upregulated in human melanomas [44]. Because CCND1 is transcriptionally regulated by MITF [45], wogonin was presumed to inhibit CCND1 expression in human melanoma cells by downregulating MITF. Therefore, wogonin is a potential therapeutic agent against melanoma.

In this study, we found that an *S*. *baicalensis* extract and its active components, wogonin and wogonoside, possessed a strong inhibitory effect on melanogenesis without cytotoxicity. In addition, *O*-methylated flavones, such as wogonin, reversibly suppress melanosome transport via MLPH downregulation. Our findings reveal the potential applicability of *S*. *baicalensis* extracts and its flavones for skin lightening and the treatment of hyperpigmentation.

## Supporting information

S1 Fig The five major flavones in *S*. *baicalensis*. (TIF)

S2 Fig The 7-*O*-methylation of baicalein mediates melanosome transport inhibition. B16F10 cells were cultured for 3 days with 50 μM baicalein or 7-*O*-methyl baicalein. (**A**) The structure of 7-*O*-methylated baicalein. (**B**) Bright field images show the melanosome distribution. Scale bar = 20 μm. (**C**) The results are expressed as the percentage of cells showing perinuclear melanosome aggregation.
(TIF)

S3 Fig Inhibitory effect of wogonin and wogonoside on melanogenesis in B16F10 melanoma cells cultured in RPMI with or without additional _L_-tyrosine. B16F10 cells were seeded in 100-mm dishes at a density of 2.0 × 10^5^ cells per dish and then cultured in RPMI supplemented with 10% FBS for 24 h. After replacing the culture medium with fresh medium with or without _L_-tyrosine (200 μM), cells were coincubated with wogonin or wogonoside (50 μM) for 3 days.
(TIF)

S4 Fig The influence of wogonin on the color of melanin in HEMs from moderately pigmented skin. HEMs from moderately pigmented skin (newborn, Thermo Fisher Scientific) were seeded in 60-mm dishes at a density of 1.0 × 10^5^ cells per dish and then incubated in Medium 254 with human melanocyte growth supplement for 24 h. After replacing the culture medium with fresh medium, cells were coincubated with 25 μM wogonin or 1 μM H-89. The culture media with or without the test compounds were changed every other day. The color and content of melanin in HEMs were determined after 7 days.
(TIF)

S5 Fig Wogonin downregulates the mRNA expression of SOX9 and moderately upregulates SOX10. After exposure to 50 μM wogonin for the indicated number of hours, the mRNA expression of Sox9 and Sox10 were quantified using qPCR and the following TaqMan Gene Expression Assays (Applied Biosystems): *Sox9* (assay ID Mm00448840_m1) and *Sox10* (assay ID Mm00569909_m1).
(TIF)

S6 Fig Wogonin downregulates the mRNA expression of OA1. After exposure to 50 μM wogonin for the indicated number of hours, the mRNA expression of OA1 was quantified using qPCR and the following TaqMan Gene Expression Assays (Applied Biosystems): *Oa1* (assay ID Mm00440553_m1).
(TIF)

S7 Fig Knockdown of MLPH induces perinuclear melanosome aggregation and a moderate reduction in melanin content in B16F10 cells. B16F10 cells were transfected with nontargeting siRNA (NT) or *Mlph* siRNA (10 nM) using a Microporator Neon (Thermo Fisher Scientific) and then cultured for 72 h. (**A**) MLPH protein expression was analyzed via immunoblotting with an antibody specific for MLPH. (**B**) *Mlph* mRNA expression was quantified using qPCR. (**C**) Bright field images show the melanosome distribution. Scale bar = 50 μm. (**D**) The level of melanogenesis was determined by quantifying the intracellular melanin content.
(TIF)

S8 Fig Multi-*O*-methyl flavones do not induce perinuclear melanosome aggregation. (**A**) The structures of two multi-*O*-methyl flavones. (**B**) B16F10 cells were cultured for 3 days with 50 μM nobiletin or 5,7-*O*-dimethyl flavone. Bright field images show the melanosome distribution. Scale bar = 10 μm.
(TIF)

S9 Fig Wogonin inhibits melanosome transport and the mRNA expression of MITF and CCND1 in human melanoma cells. (**A**–**B**) MM–ANs were cultured for 3 days with 25 μM wogonin. Melanosomes were immunostained with anti-TYRP1 or anti-HMB45 antibodies and visualized with an Alexa Fluor 488 secondary antibody. Nuclei and F-actin were stained with DAPI and phalloidin with a fluorescent analog (Alexa Fluor 594), respectively. Nuclei and F-actin fluorescence and immunostaining for melanosomes were analyzed by confocal microscopy. Scale bar = 10 μm. (**C**–**D**) After exposure to 25 μM wogonin for 24 h, the mRNA expression of MITF and CCND1 was quantified using qPCR.
(TIF)

S1 Text Supporting Materials and Methods; Supporting References. (DOCX)

## References

[1] Lin JY, Fisher DE. Melanocyte biology and skin pigmentation. Nature. 2007;445: 843–850. 10.1038/nature05660 17314970

[2] Kondo T, Hearing VJ. Update on the regulation of mammalian melanocyte function and skin pigmentation. Expert Rev Dermatol. 2011;6: 97–108. 10.1586/edm.10.70 21572549 PMC3093193

[3] Hearing VJ. Determination of melanin synthetic pathways. J Invest Dermatol. 2011;131: E8–E11. 10.1038/skinbio.2011.4 22094404 PMC6944209

[4] Kuroda TS, Fukuda M. Rab27A-binding protein Slp2-a is required for peripheral melanosome distribution and elongated cell shape in melanocytes. Nat Cell Biol. 2004;6: 1195–1203. 10.1038/ncb1197 15543135

[5] Wu XS, Rao K, Zhang H, Wang F, Sellers JR, Matesic LE, et al Identification of an organelle receptor for myosin-Va. Nat Cell Biol. 2002;4: 271–278. 10.1038/ncb760 11887186

[6] Mercer JA, Seperack PK, Strobel MC, Copeland NG, Jenkins NA. Novel myosin heavy chain encoded by murine dilute coat colour locus. Nature. 1991;349: 709–713. 10.1038/349709a0 1996138

[7] Matesic LE, Yip R, Reuss AE, Swing DA, O'Sullivan TN, Fletcher CF, et al Mutations in Mlph, encoding a member of the Rab effector family, cause the melanosome transport defects observed in leaden mice. Proc Natl Acad Sci U S A. 2001;98: 10238–10243. 10.1073/pnas.181336698 11504925 PMC56945

[8] Wilson SM, Yip R, Swing DA, O'Sullivan TN, Zhang Y, Novak EK, et al A mutation in Rab27a causes the vesicle transport defects observed in ashen mice. Proc Natl Acad Sci U S A. 2000;97: 7933–7938. 10.1073/pnas.140212797 10859366 PMC16648

[9] Wu X, Zhang H, Salmani JM, Fu R, Chen B. Advances of wogonin, an extract from Scutellaria baicalensis, for the treatment of multiple tumors. Onco Targets Ther. 2016;9: 2935–2943. 10.2147/OTT.S105586 27274287 PMC4876109

[10] Xiao JR, Do CW, To CH. Potential therapeutic effects of baicalein, baicalin, and wogonin in ocular disorders. J Ocul Pharmacol Ther. 2014;30: 605–614. 10.1089/jop.2014.0074 25280175

[11] Li C, Lin G, Zuo Z. Pharmacological effects and pharmacokinetics properties of Radix Scutellariae and its bioactive flavones. Biopharm Drug Dispos. 2011;32: 427–445. 10.1002/bdd.771 21928297

[12] Tsuji-Naito K, Hatani T, Okada T, Tehara T. Modulating effects of a novel skin-lightening agent, alpha-lipoic acid derivative, on melanin production by the formation of DOPA conjugate products. Bioorg Med Chem. 2007; 15: 1967–1975. 10.1016/j.bmc.2006.12.042 17218103

[13] Murase D, Hachiya A, Takano K, Hicks R, Visscher MO, Kitahara T, et al Autophagy has a significant role in determining skin color by regulating melanosome degradation in keratinocytes. J Invest Dermatol. 2013;133: 2416–2424. 10.1038/jid.2013.165 23558403

[14] Julkunen-Tiitto R. Phenolic constituents in the leaves of northern willows: Methods for the analysis of certain phenolics. J Agric Food Chem. 1985;33: 213–217.

[15] Gálvez M, Martín-Cordero C, Houghton PJ, Ayuso MJ. Antioxidant activity of methanol extracts obtained from Plantago species. J Agric Food Chem. 2005;53: 1927–1933. 10.1021/jf048076s 15769115

[16] Kuroda TS, Ariga H, Fukuda M. The actin-binding domain of Slac2-a/melanophilin is required for melanosome distribution in melanocytes. Mol Cell Biol. 2003;23: 5245–5255. 10.1128/MCB.23.15.5245-5255.2003 12861011 PMC165717

[17] Ando H, Watabe H, Valencia JC, Yasumoto K, Furumura M, Funasaka Y, et al Fatty acids regulate pigmentation via proteasomal degradation of tyrosinase: a new aspect of ubiquitin-proteasome function. J Biol Chem. 2004;279: 15427–15433. 10.1074/jbc.M313701200 14739285

[18] Liu XF, Liu ML, Iyanagi T, Legesse K, Lee TD, Chen SA. Inhibition of rat liver NAD(P)H:quinone acceptor oxidoreductase (DT-diaphorase) by flavonoids isolated from the Chinese herb scutellariae radix (Huang Qin). Mol Pharmacol. 1990;37: 911–915. 1694261

[19] Cheli Y, Luciani F, Khaled M, Beuret L, Bille K, Gounon P, et al {alpha}MSH and Cyclic AMP elevating agents control melanosome pH through a protein kinase A-independent mechanism. J Biol Chem. 2009;284: 18699–18706. 10.1074/jbc.M109.005819 19389708 PMC2707200

[20] Schepsky A, Bruser K, Gunnarsson GJ, Goodall J, Hallsson JH, Goding CR, et al The microphthalmia-associated transcription factor Mitf interacts with beta-catenin to determine target gene expression. Mol Cell Biol. 2006;26: 8914–8927. 10.1128/MCB.02299-05 17000761 PMC1636837

[21] Zhao K, Wei L, Hui H, Dai Q, You QD, Guo QL, et al Wogonin suppresses melanoma cell B16-F10 invasion and migration by inhibiting Ras-medicated pathways. PLoS One. 2014;9: e106458 10.1371/journal.pone.0106458 25203554 PMC4159230

[22] Hammer JA III, Sellers JR. Walking to work: roles for class V myosins as cargo transporters. Nat Rev Mol Cell Biol. 2011;13: 13–26. 10.1038/nrm3248 22146746

[23] Fukuda M, Itoh T. Slac2-a/melanophilin contains multiple PEST-like sequences that are highly sensitive to proteolysis. J Biol Chem. 2004;279: 22314–22321. 10.1074/jbc.401791200 15145961

[24] Wasmeier C, Romao M, Plowright L, Bennett DC, Raposo G, Seabra MC. Rab38 and Rab32 control post-Golgi trafficking of melanogenic enzymes. J Cell Biol. 2006;175: 271–281. 10.1083/jcb.200606050 17043139 PMC2064568

[25] Slominski A, Zmijewski MA, Pawelek J. L-tyrosine and L-dihydroxyphenylalanine as hormone-like regulators of melanocyte functions. Pigment Cell Melanoma Res. 2012; 25: 14–27. 10.1111/j.1755-148X.2011.00898.x 21834848 PMC3242935

[26] Wolnicka-Glubisz A, Nogal K, Żądło A, Płonka PM. Curcumin does not switch melanin synthesis towards pheomelanin in B16F10 cells. Arch Dermatol Res. 2015; 307: 89–98. 10.1007/s00403-014-1523-1 25398276

[27] Slominski A, Tobin DJ, Shibahara S, Wortsman J. Melanin pigmentation in mammalian skin and its hormonal regulation. Physiol Rev. 2004; 84: 1155–1228. 10.1152/physrev.00044.2003 15383650

[28] Bennett DC, Huszar D, Laipis PJ, Jaenisch R, Jackson IJ. Phenotypic rescue of mutant brown melanocytes by a retrovirus carrying a wild-type tyrosinase-related protein gene. Development. 1990; 110: 471–475. 2133550 10.1242/dev.110.2.471

[29] D'Mello SA, Finlay GJ, Baguley BC, Askarian-Amiri ME. Signaling Pathways in Melanogenesis. Int J Mol Sci. 2016; 17: E1144 10.3390/ijms17071144 27428965 PMC4964517

[30] Vachtenheim J, Borovanský J. "Transcription physiology" of pigment formation in melanocytes: central role of MITF. Exp Dermatol. 2010; 19: 617–627. 10.1111/j.1600-0625.2009.01053.x 20201954

[31] Itakura K, Takayama A, Kondo C, Fukuda M, inventor; KOSÉ Corp., applicant. Slp-2a protein reducers and Myosin-5a protein reducers. Japanese Patent 5507866. 2014 Mar 28.

[32] Hata Y, Itakura K, Takayama A, Kondo C, Fukuda M. Japan Patent Kokai. 2009; 2009–294615.

[33] Chiaverini C, Beuret L, Flori E, Busca R, Abbe P, Bille K, et al Microphthalmia-associated transcription factor regulates RAB27A gene expression and controls melanosome transport. J Biol Chem. 2008;283: 12635–12642. 10.1074/jbc.M800130200 18281284

[34] Hoek KS, Schlegel NC, Eichhoff OM, Widmer DS, Praetorius C, Einarsson SO, et al Novel MITF targets identified using a two-step DNA microarray strategy. Pigment Cell Melanoma Res. 2008; 21: 665–676. 10.1111/j.1755-148X.2008.00505.x 19067971

[35] Palmisano I, Bagnato P, Palmigiano A, Innamorati G, Rotondo G, Altimare D, et al The ocular albinism type 1 protein, an intracellular G protein-coupled receptor, regulates melanosome transport in pigment cells. Hum Mol Genet. 2008; 17: 3487–3501. 10.1093/hmg/ddn241 18697795 PMC2572695

[36] Ménasché G, Ho CH, Sanal O, Feldmann J, Tezcan I, Ersoy F, et al Griscelli syndrome restricted to hypopigmentation results from a melanophilin defect (GS3) or a MYO5A F-exon deletion (GS1). J Clin Invest. 2003;112: 450–456. 10.1172/JCI18264 12897212 PMC166299

[37] Chang H, Choi H, Joo KM, Kim D, Lee TR. Manassantin B inhibits melanosome transport in melanocytes by disrupting the melanophilin-myosin Va interaction. Pigment Cell Melanoma Res. 2012;25: 765–772. 10.1111/pcmr.12002 22863119

[38] Kang YG, Choi EJ, Choi Y, Hwang JK. 5,7-dimethoxyflavone induces melanogenesis in B16F10 melanoma cells through cAMP-dependent signalling. Exp Dermatol. 2011;20: 445–447. 10.1111/j.1600-0625.2010.01236.x 21426409

[39] Cao G, Sofic E, Prior RL. Antioxidant and prooxidant behavior of flavonoids: structure-activity relationships. Free Radic Biol Med. 1997;22: 749–760. 9119242 10.1016/s0891-5849(96)00351-6

[40] Gao Z, Huang K, Yang X, Xu H. Free radical scavenging and antioxidant activities of flavonoids extracted from the radix of Scutellaria baicalensis Georgi. Biochim Biophys Acta. 1999;1472: 643–650. 10564778 10.1016/s0304-4165(99)00152-x

[41] Brożyna AA, Jóźwicki W, Carlson JA, Slominski AT. Melanogenesis affects overall and disease-free survival in patients with stage III and IV melanoma. Hum Pathol. 2013; 44: 2071–2074. 10.1016/j.humpath.2013.02.022 23791398 PMC3783651

[42] Zhao K, Wei L, Hui H, Dai Q, You QD, Guo QL, et al Wogonin suppresses melanoma cell B16-F10 invasion and migration by inhibiting Ras-medicated pathways. PLoS One. 2014; 9: e106458 10.1371/journal.pone.0106458 25203554 PMC4159230

[43] Garraway LA, Widlund HR, Rubin MA, Getz G, Berger AJ, Ramaswamy S, et al Integrative genomic analyses identify MITF as a lineage survival oncogene amplified in malignant melanoma. Nature. 2005; 436: 117–122. 10.1038/nature03664 16001072

[44] Yajima I, Kumasaka MY, Thang ND, Goto Y, Takeda K, Iida M, et al Molecular network associated with MITF in skin melanoma development and progression. J Skin Cancer. 2011; 2011: 730170 10.1155/2011/730170 22046555 PMC3199194

[45] Pan L, Ma X, Wen B, Su Z, Zheng X, et al Microphthalmia-associated transcription factor/T-box factor-2 axis acts through Cyclin D1 to regulate melanocyte proliferation. Cell Prolif. 2015; 48: 631–642. 10.1111/cpr.12227 26486273 PMC6496872

