# Supplementary figures and images for: Bifunctional effects of O-methylated flavones from Scutellaria baicalensis Georgi on melanocytes: Inhibition of melanin production and intracellular melanosome transport
Source: PLoS One. 2017 Feb 9;12(2):e0171513. doi: 10.1371/journal.pone.0171513 (PMC5300169; doi:10.1371/journal.pone.0171513)

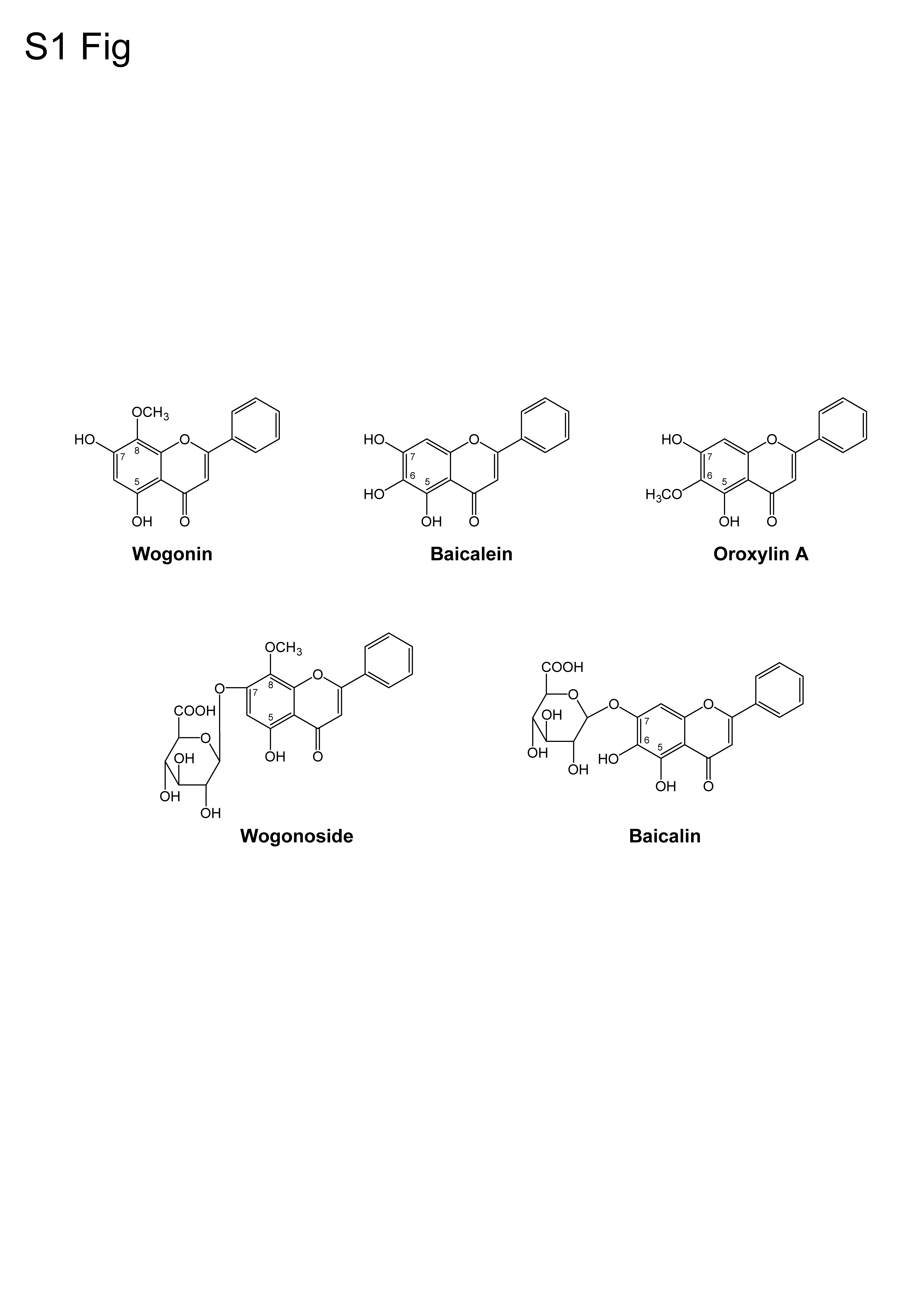

Supplement: S1 Fig — (TIF) [file pone.0171513.s001.tif]

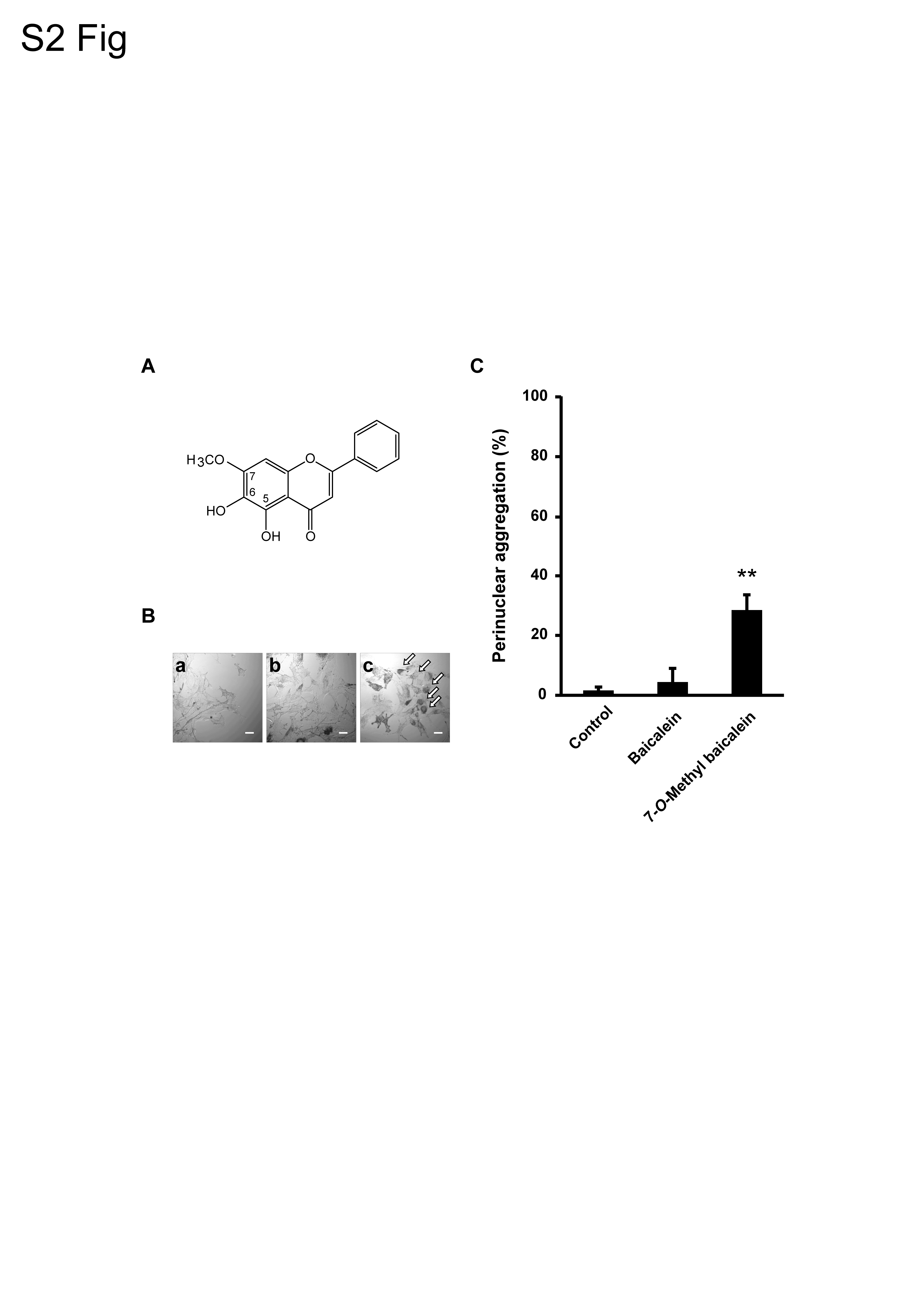

Supplement: S2 Fig — B16F10 cells were cultured for 3 days with 50 μM baicalein or 7-O-methyl baicalein. (A) The structure of 7-O-methylated baicalein. (B) Bright field images show the melanosome distribution. Scale bar = 20 μm. (C) The results are expressed as the percentage of cells showing perinuclear melanosome aggregation. (TIF) [file pone.0171513.s002.tif]

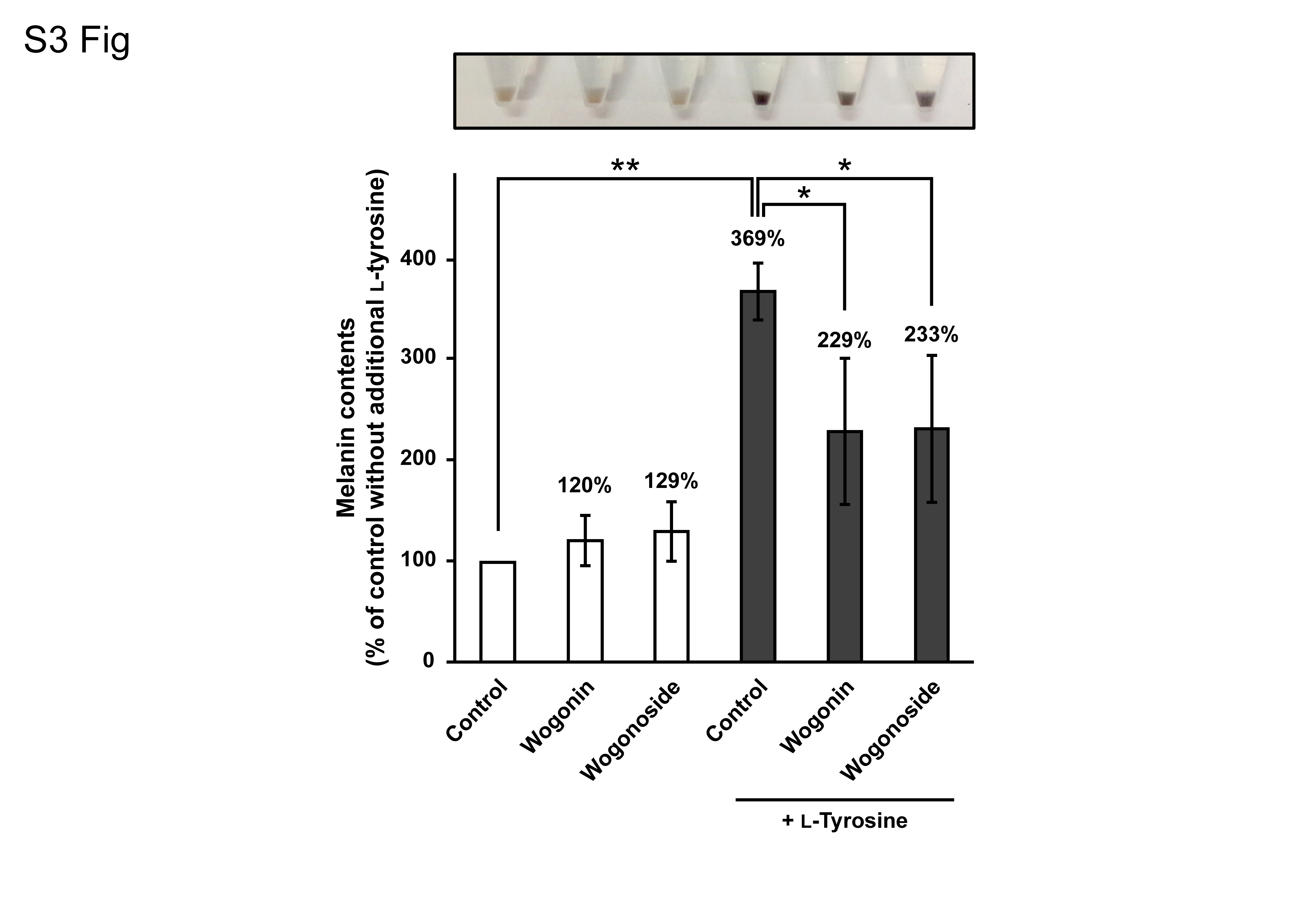

Supplement: S3 Fig — B16F10 cells were seeded in 100-mm dishes at a density of 2.0 × 10^5^ cells per dish and then cultured in RPMI supplemented with 10% FBS for 24 h. After replacing the culture medium with fresh medium with or without L-tyrosine (200 μM), cells were coincubated with wogonin or wogonoside (50 μM) for 3 days. (TIF) [file pone.0171513.s003.tif]

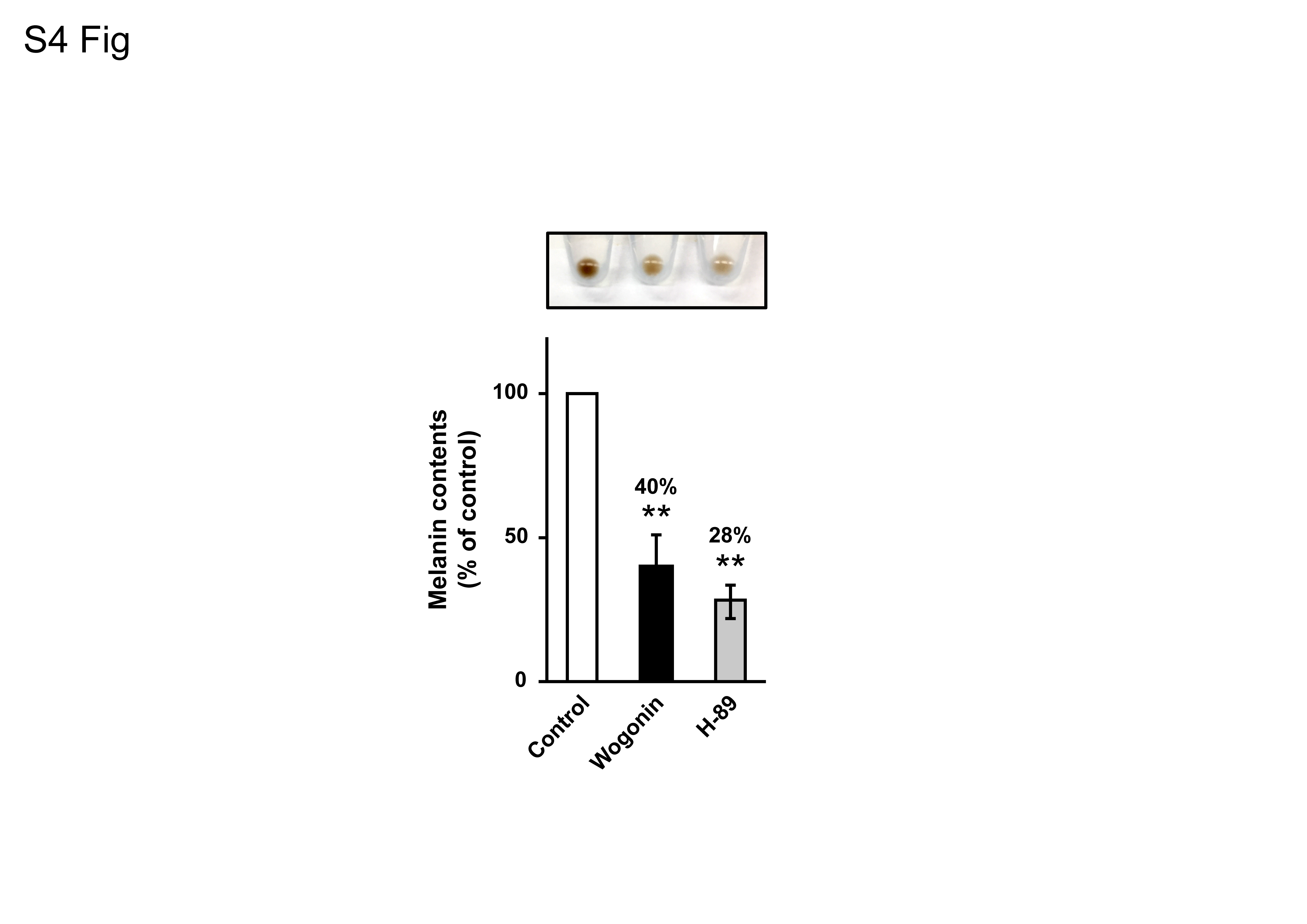

Supplement: S4 Fig — HEMs from moderately pigmented skin (newborn, Thermo Fisher Scientific) were seeded in 60-mm dishes at a density of 1.0 × 10^5^ cells per dish and then incubated in Medium 254 with human melanocyte growth supplement for 24 h. After replacing the culture medium with fresh medium, cells were coincubated with 25 μM wogonin or 1 μM H-89. The culture media with or without the test compounds were changed every other day. The color and content of melanin in HEMs were determined after 7 days. (TIF) [file pone.0171513.s004.tif]

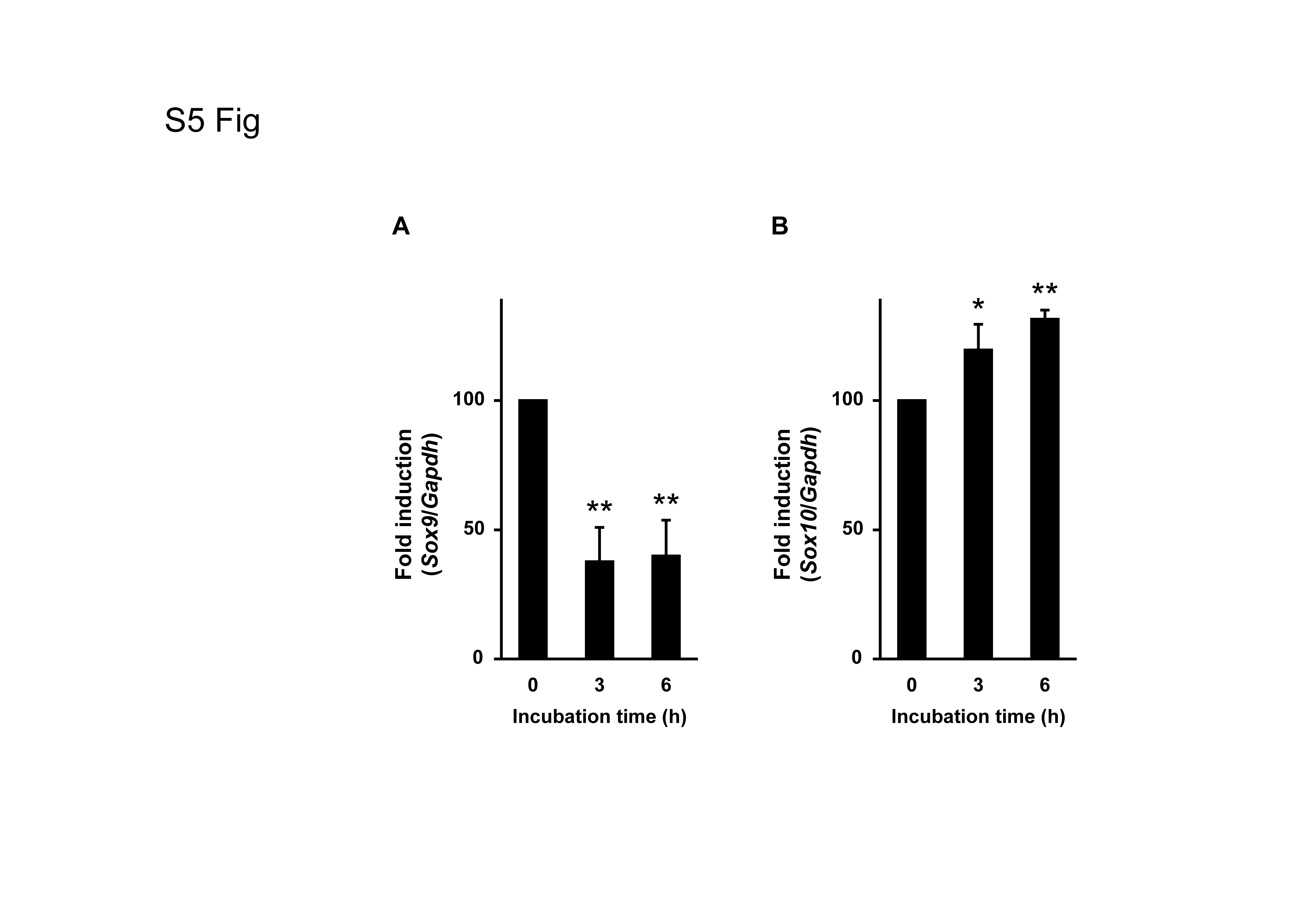

Supplement: S5 Fig — After exposure to 50 μM wogonin for the indicated number of hours, the mRNA expression of Sox9 and Sox10 were quantified using qPCR and the following TaqMan Gene Expression Assays (Applied Biosystems): Sox9 (assay ID Mm00448840_m1) and Sox10 (assay ID Mm00569909_m1). (TIF) [file pone.0171513.s005.tif]

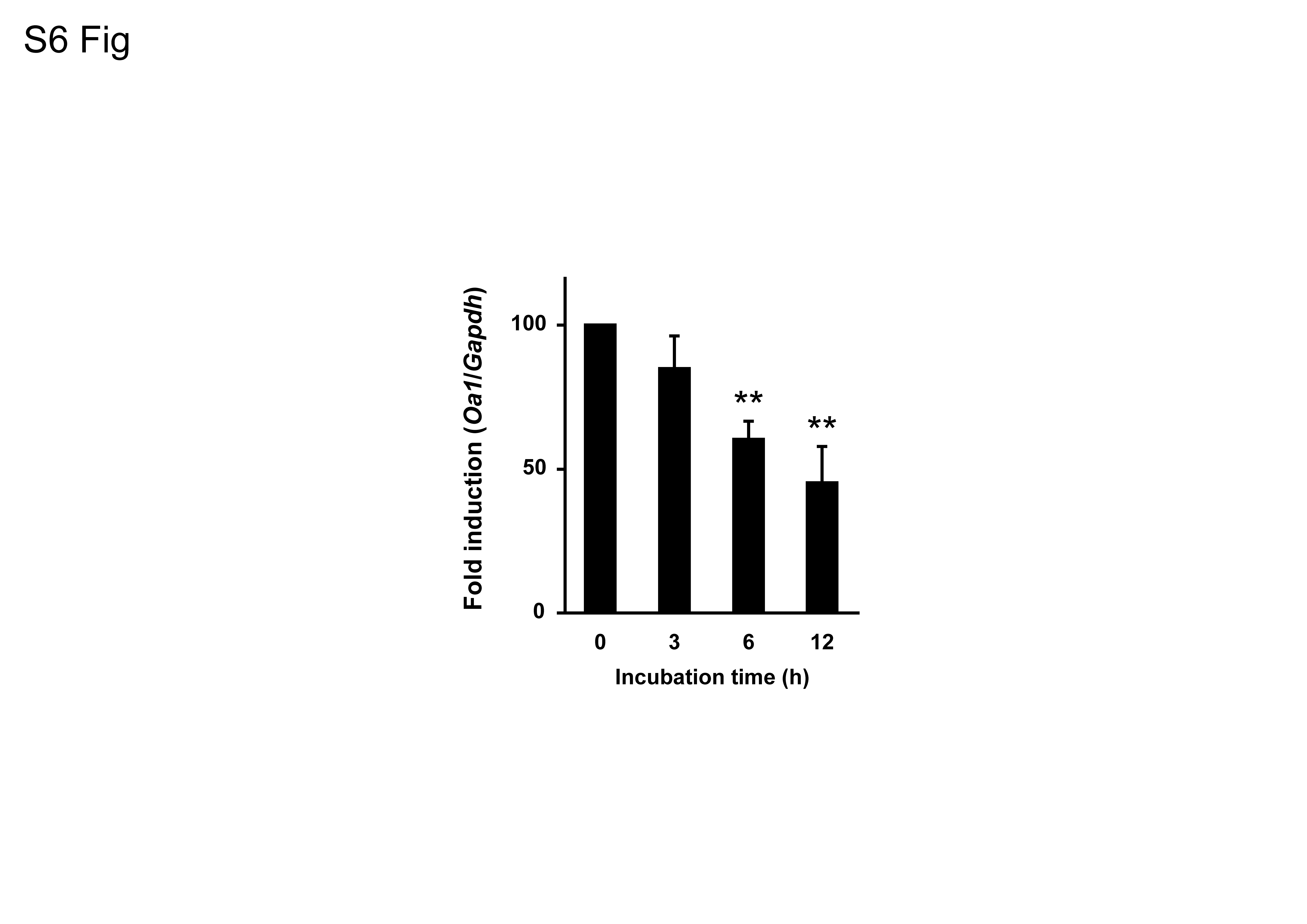

Supplement: S6 Fig — After exposure to 50 μM wogonin for the indicated number of hours, the mRNA expression of OA1 was quantified using qPCR and the following TaqMan Gene Expression Assays (Applied Biosystems): Oa1 (assay ID Mm00440553_m1). (TIF) [file pone.0171513.s006.tif]

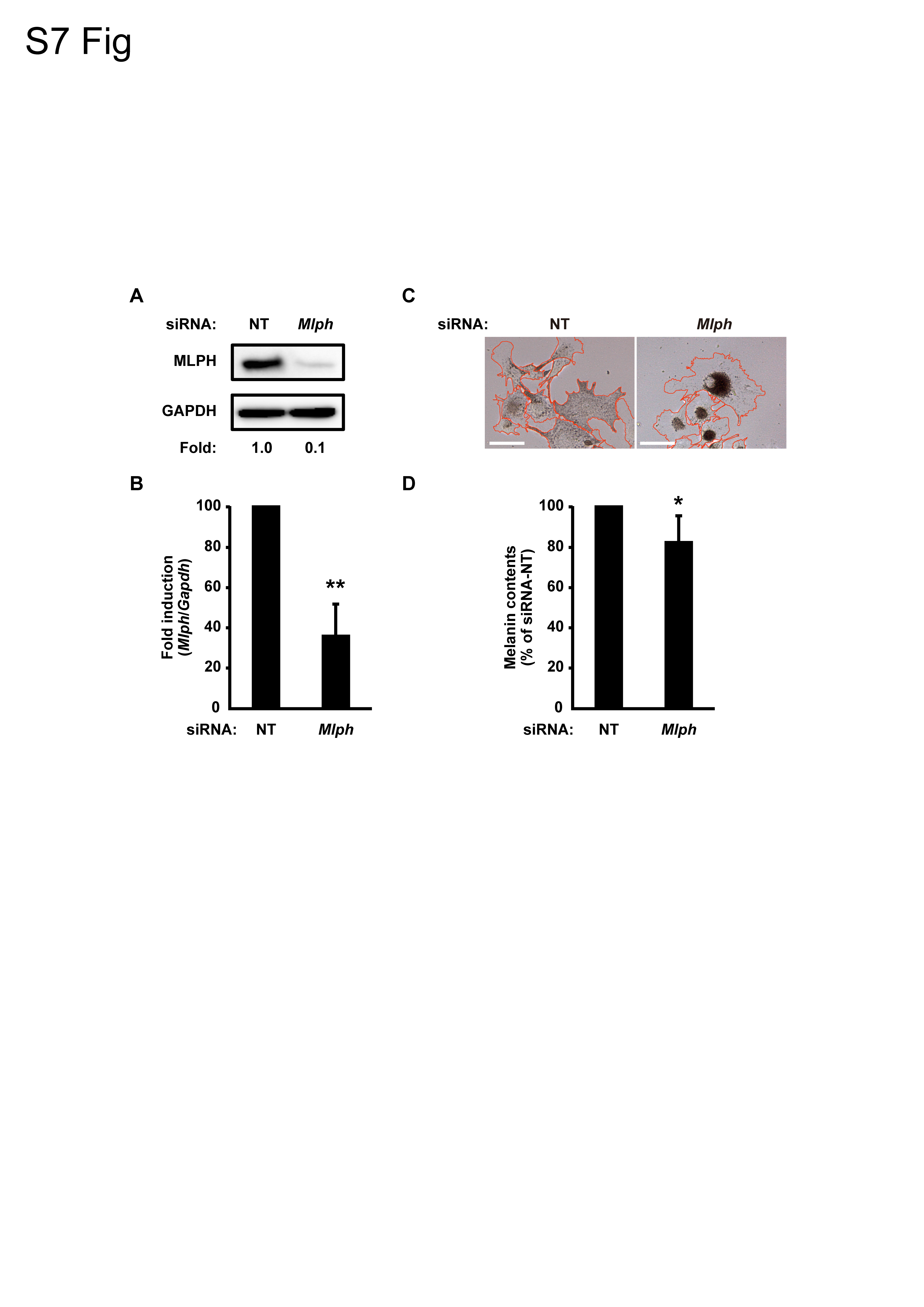

Supplement: S7 Fig — B16F10 cells were transfected with nontargeting siRNA (NT) or Mlph siRNA (10 nM) using a Microporator Neon (Thermo Fisher Scientific) and then cultured for 72 h. (A) MLPH protein expression was analyzed via immunoblotting with an antibody specific for MLPH. (B) Mlph mRNA expression was quantified using qPCR. (C) Bright field images show the melanosome distribution. Scale bar = 50 μm. (D) The level of melanogenesis was determined by quantifying the intracellular melanin content. (TIF) [file pone.0171513.s007.tif]

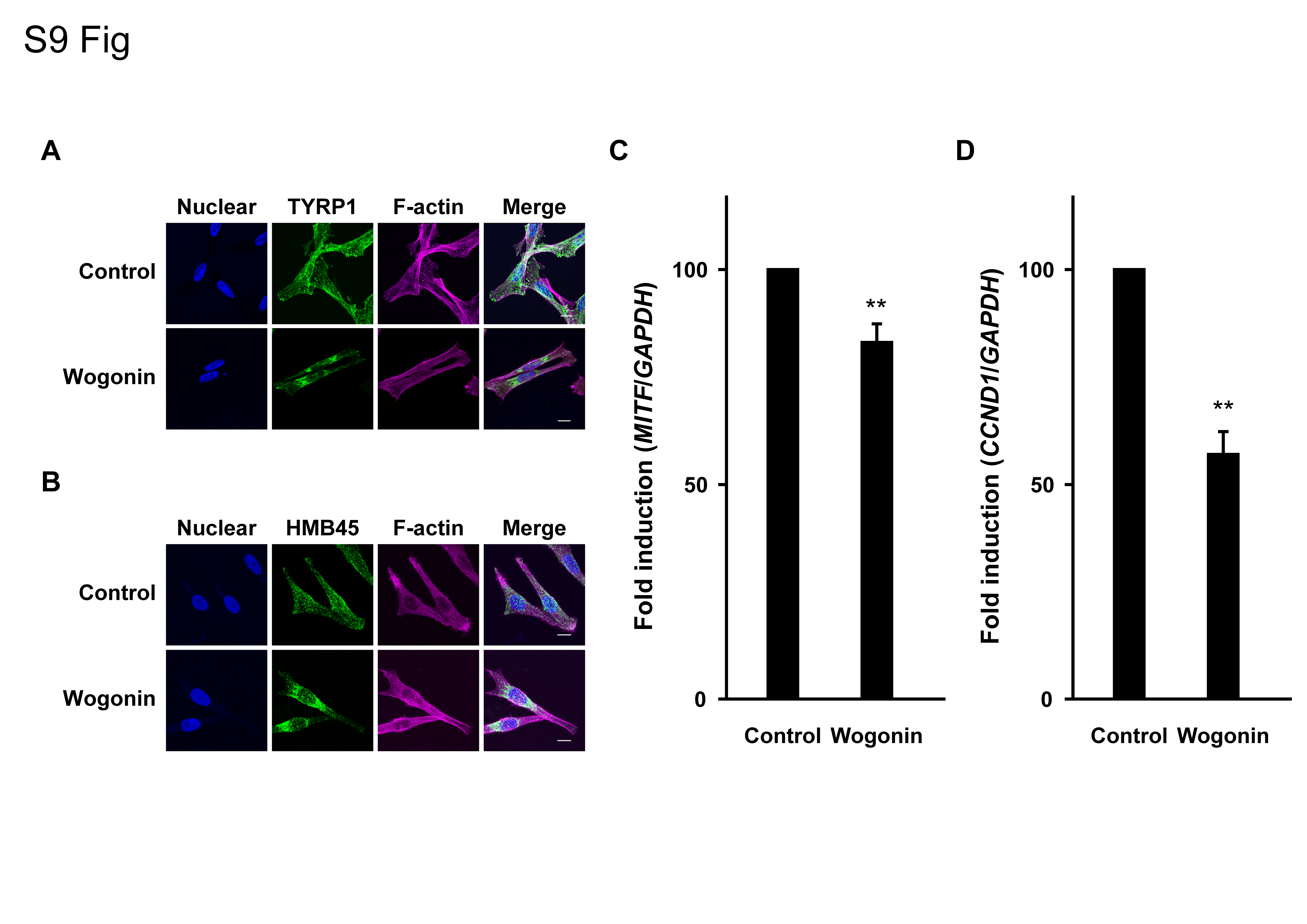

Supplement: S9 Fig — (A–B) MM–ANs were cultured for 3 days with 25 μM wogonin. Melanosomes were immunostained with anti-TYRP1 or anti-HMB45 antibodies and visualized with an Alexa Fluor 488 secondary antibody. Nuclei and F-actin were stained with DAPI and phalloidin with a fluorescent analog (Alexa Fluor 594), respectively. Nuclei and F-actin fluorescence and immunostaining for melanosomes were analyzed by confocal microscopy. Scale bar = 10 μm. (C–D) After exposure to 25 μM wogonin for 24 h, the mRNA expression of MITF and CCND1 was quantified using qPCR. (TIF) [file pone.0171513.s009.tif]
