# Supplementary material for: Bifunctional effects of O-methylated flavones from Scutellaria baicalensis Georgi on melanocytes: Inhibition of melanin production and intracellular melanosome transport
Source: PLoS One. 2017 Feb 9;12(2):e0171513. doi: 10.1371/journal.pone.0171513 (PMC5300169; doi:10.1371/journal.pone.0171513)
Supplement: S1 Text — (DOCX) [file pone.0171513.s010.docx]

**Supporting Information**

**S1 Materials and Methods Appendix**

**Materials**

Anti-human melanoma black (HMB45) (ab787) antibody was purchased from Abcam. Alexa Fluor 488-conjugated anti-mouse antibody was obtained from Thermo Fisher Scientific.

**Cell cultures**

The human malignant melanoma cell line MM–AN was a gift from Dr. Toshihide Akasaka (Iwate Medical University, Iwate, Japan). MM­–AN cells were derived from the inguinal region of patient AN [1, 2]. MM–AN cells were maintained as monolayer cultures in RPMI 1640 medium (RPMI) supplemented with 10% fetal bovine serum (FBS) at 37°C in a humidified atmosphere containing 5% CO_2_.

**Immunofluorescence analysis**

MM–AN cells were seeded in 35-mm dishes with preinserted collagen coated coverslips at a density of 2.5 × 10^4^ cells per dish and then cultured in RPMI supplemented with 10% FBS for 24 h. After replacing the culture medium with fresh medium, cells were coincubated with wogonin (25 µM) for 3 days. Cells on coverslips were fixed for 15 min in 4% paraformaldehyde, permeabilized with 0.1% Triton-X 100 for 10 min, and incubated in a blocking solution (3% BSA in PBS) for 1 h. To detect actin, cells were treated with diluted Alexa Fluor 594 phalloidin for 20 min. For HMB45 coimmunostaining, cells were exposed to anti-HMB45 (1:250 dilution) antibodies in PBS containing 1% BSA for 16 h at 4°C and subsequently incubated with Alexa Fluor 488-conjugated anti-mouse antibodies (1:400 dilution) for 1 h. For nuclear counterstaining, cells were treated with 1 µg/ml DAPI. Mounted samples were observed using a TCS SP2 AOBS confocal laser microscope (Leica Microsystems).

**Quantitative PCR (qPCR)**

MM–AN cells were seeded in 6-well plates at a density of 1.0 × 10^5^ cells per well and then cultured in RPMI supplemented with 10% FBS for 24 h. After replacing the culture medium with fresh RPMI medium supplemented with 2% FBS, cells were coincubated with wogonin (25 µM) for 24 h. Total RNA was isolated from cultured cells using an RNeasy Mini Kit (Qiagen) per the manufacturer’s instructions. RNA was stored in RNase-free water at −80°C prior to reverse transcription. First-strand cDNA was synthesized with 1 μg of total RNA using a PrimeScript II 1st strand cDNA Synthesis Kit (Takara Bio) per the manufacturer’s instructions. mRNA expression levels of target genes were measured using an Applied Biosystems 7500 Real Time PCR System (Applied Biosystems) and the following TaqMan Gene Expression Assays: *MITF* (assay ID Hs01117294_m1); CCND1 (assay ID Hs00765553_m1); and *GAPDH* (assay ID Hs02758991_g1). All reactions were performed in triplicate. *GAPDH* was used as a housekeeping gene for normalization. The relative amount of mRNA was calculated using the comparative C_T_ method.

**Statistical analyses**

The data are expressed as the mean ± SD from at least three independent experiments. Statistical analyses were performed using the Tukey–Kramer test. Asterisks indicate statistical significance when compared with the control group (***p* < 0.01).

**References**

1. Byers HR, Etoh T, Doherty JR, Sober AJ, Mihm MC Jr. Cell migration and actin organization in cultured human primary, recurrent cutaneous and metastatic melanoma. Time-lapse and image analysis. Am J Pathol. 1991; 139: 423-435.
2. Uppada SB, Erickson T, Wojdyla L, Moravec DN, Song Z, et al. Novel delivery system for T-oligo using a nanocomplex formed with an alpha helical peptide for melanoma therapy. Int J Nanomedicine. 2014; 9: 43-53.
